# HER2 Phosphorylation Is Maintained by a PKB Negative Feedback Loop in Response to Anti-HER2 Herceptin in Breast Cancer

**DOI:** 10.1371/journal.pbio.1000563

**Published:** 2010-12-21

**Authors:** Merel Gijsen, Peter King, Tim Perera, Peter J. Parker, Adrian L. Harris, Banafshé Larijani, Anthony Kong

**Affiliations:** 1 Molecular Oncology Laboratories, Weatherall Institute of Molecular Medicine, University of Oxford, John Radcliffe Hospital, Oxford, United Kingdom; 2 Oncology Discovery Research and Early Development, Johnson & Johnson Pharmaceutical Research and Development, Janssen Pharmaceutica, Beerse, Belgium; 3 Protein Phosphorylation Laboratory, London Research Institute, Cancer Research UK, London, United Kingdom; 4 Division of Cancer Studies, King's College School of Medicine, Guy's Hospital, London, United Kingdom; 5 Cell Biophysics Laboratory, London Research Institute, Cancer Research UK, London, United Kingdom; Fred Hutchinson Cancer Research Center, United States of America

## Abstract

Herceptin (trastuzumab) is used in patients with breast cancer who have HER2 (ErbB2)–positive tumours. However, its mechanisms of action and how acquired resistance to Herceptin occurs are still poorly understood. It was previously thought that the anti-HER2 monoclonal antibody Herceptin inhibits HER2 signalling, but recent studies have shown that Herceptin does not decrease HER2 phosphorylation. Its failure to abolish HER2 phosphorylation may be a key to why acquired resistance inevitably occurs for all responders if Herceptin is given as monotherapy. To date, no studies have explained why Herceptin does not abolish HER2 phosphorylation. The objective of this study was to investigate why Herceptin did not decrease HER2 phosphorylation despite being an anti-HER2 monoclonal antibody. We also investigated the effects of acute and chronic Herceptin treatment on HER3 and PKB phosphorylation in HER2-positive breast cancer cells. Using both Förster resonance energy transfer (FRET) methodology and conventional Western blot, we have found the molecular mechanisms whereby Herceptin fails to abolish HER2 phosphorylation. HER2 phosphorylation is maintained by ligand-mediated activation of EGFR, HER3, and HER4 receptors, resulting in their dimerisation with HER2. The release of HER ligands was mediated by ADAM17 through a PKB negative feedback loop. The feedback loop was activated because of the inhibition of PKB by Herceptin treatment since up-regulation of HER ligands and ADAM17 also occurred when PKB phosphorylation was inhibited by a PKB inhibitor (Akt inhibitor VIII, Akti-1/2). The combination of Herceptin with ADAM17 inhibitors or the panHER inhibitor JNJ-26483327 was able to abrogate the feedback loop and decrease HER2 phosphorylation. Furthermore, the combination of Herceptin with JNJ-26483327 was synergistic in tumour inhibition in a BT474 xenograft model. We have determined that a PKB negative feedback loop links ADAM17 and HER ligands in maintaining HER2 phosphorylation during Herceptin treatment. The activation of other HER receptors via ADAM17 may mediate acquired resistance to Herceptin in HER2-overexpressing breast cancer. This finding offers treatment opportunities for overcoming resistance in these patients. We propose that Herceptin should be combined with a panHER inhibitor or an ADAM inhibitor to overcome the acquired drug resistance for patients with HER2-positive breast cancer. Our results may also have implications for resistance to other therapies targeting HER receptors.

## Abstract 2

A feedback loop maintains HER2 receptor signalling and cell survival in response to Herceptin treatment in HER2-positive breast cancers, but this Herceptin resistance may be bypassed by pan-HER inhibitors.

## Author Summary

HER2 (ErbB2) is a surface protein and member of the epidermal growth factor receptor (EGFR) family that is overexpressed in approximately one-fifth of breast cancers. HER2-positive breast tumours tend to be very aggressive, and patients with this type of tumour have a poor prognosis. A therapeutic monoclonal antibody called trastuzumab (Herceptin) has been designed to block HER2 signalling and is used as a treatment for patients with HER2-positive breast cancer. However, recent studies have shown that Herceptin does not decrease HER2 activation. This may be why patients invariably develop resistance if treated with Herceptin monotherapy. To date, no study has explained why Herceptin cannot abolish HER2 signalling despite being an anti-HER2 monoclonal antibody. We have found that Herceptin switches on a feedback loop that increases the production of the ADAM17 protein, a protease that in turn releases the growth factors that activate HER (ErbB) receptors. These growth factors activate HER2 and also the other members of the HER receptor family—EGFR, HER3 and HER4—in such a way as to maintain HER2 activation and cell survival in HER2-positive breast cancer cells. We have found that when Herceptin is provided in combination with ADAM17 inhibitors, the feedback loop is abrogated in cells. Furthermore, a pan-HER inhibitor that decreases the activation of other HER receptors can also inhibit the feedback loop and decrease HER2 activation when used in combination with Herceptin. We further demonstrated that the combination therapy of Herceptin with a pan-HER inhibitor is more effective than Herceptin alone in an animal model of breast cancer. We believe our results offer treatment strategies that may help overcome acquired Herceptin resistance in patients with HER2-positive breast cancer.

## Introduction

Dysregulation of human epidermal growth factor (HER/ErbB) receptors is implicated in various epithelial cancers [1]. The four HER receptors are capable of dimerising with each other (homodimerisation) or with different HER receptors (heterodimerisation) upon ligand binding [2]. The homo- or heterodimerisation of the receptors results in the activation of the intrinsic tyrosine kinase domain and autophosphorylation of specific tyrosine residues in the C-terminal tail [2]. The ligand-induced HER receptor dimerisation follows a strict hierarchy, and HER2 has been shown to be the preferred dimerisation partner [3]. The crystal structure explains why HER2 is ligandless, since its extracellular domain is always in the “open” conformation, with the projection of domain II ready for dimerisation even when monomeric [4]. This fixed “open” conformation of HER2 in the absence of ligand binding (mimicking the ligand-bound form in the EGFR structure) may account for why it is the preferred dimerisation partner [3].

Herceptin (trastuzumab) is a humanised mouse monoclonal antibody 4D5 and binds to the juxtamembrane region of HER2 of domain IV [4]. However, the precise mechanisms of its action and its acquired resistance are still poorly understood. Around 15%–20% of patients with breast cancer have HER2-positive tumours, and the amplification or overexpression of HER2 has been shown to be a significant predictor for both overall survival and time to relapse in these patients [5]. Herceptin has been shown to induce tumour regression in about a third of patients with metastatic HER2-positive breast cancer, but the response is rarely sustained if Herceptin is given as a single agent [6]. Therefore, understanding the mechanisms of its acquired resistance is of paramount importance.

The current proposed primary mechanisms of action for Herceptin include HER2 receptor down-regulation and inhibition of aberrant receptor tyrosine kinase activity [7],[8]. There is strong evidence of an immune-mediated mechanism in which the interaction of Herceptin's human Fc region with immune effector cells results in the stimulation of natural killer cells and activation of antibody-dependent cellular cytotoxicity [9],[10]. Other proposed mechanisms of Herceptin's action include inhibition of basal and activated HER2 ectodomain cleavage in breast cancer cells [11], the induction of G1 arrest and cyclin-dependent kinase inhibitor p27^Kip1^ levels [12], or activation of PTEN [13].

Although Herceptin was developed to target the HER2 receptor, recent studies have shown that Herceptin does not decrease HER2 phosphorylation [14],[15]. Its failure to abolish HER2 phosphorylation may be a key to why acquired resistance inevitably occurs for all responders if Herceptin is given as monotherapy. To date, no studies have explained why Herceptin does not abolish HER2 phosphorylation. The objective of our study was to investigate why Herceptin did not decrease HER2 phosphorylation despite being an anti-HER2 monoclonal antibody. We also investigated the effects of acute and chronic Herceptin treatment on HER3 and PKB phosphorylation in HER2-positive breast cancer cells.

We showed that HER2 phosphorylation was maintained by the activation of other HER receptors during Herceptin treatment through an ADAM17-mediated ligand release. Although Herceptin initially decreased HER3 phosphorylation, reactivation of HER3 occurred in prolonged Herceptin treatment through a PKB negative feedback loop. The reactivation of HER3 and failure of Herceptin to abolish HER2 phosphorylation may be responsible for acquired resistance to Herceptin in HER2-overexpressing breast cancer.

## Results

### Herceptin Does Not Abolish HER2 Phosphorylation in HER2-Overexpressing Breast Cancer Cells

We investigated how binding of HER2 receptors by the anti-HER2 monoclonal antibody Herceptin affects HER2 receptors. Although Herceptin was initially thought to inhibit aberrant HER2 receptor tyrosine kinase activity, recent studies have shown that Herceptin does not decrease HER2 phosphorylation [14],[15]. However, the mechanisms of why Herceptin does not inhibit HER2 phosphorylation have not been elucidated. Furthermore, studies that have investigated the effect of Herceptin on HER2 phosphorylation have typically been based on classical Western blot analysis, which cannot detect phosphorylation status in individual cells. We proceeded to monitor the effect of Herceptin on HER2 phosphorylation in HER2-overexpressing cells using classical biochemical methods in combination with an established Förster resonance energy transfer (FRET) methodology that can assess HER2 phosphorylation in individual cells [16].

**Figure 1:**
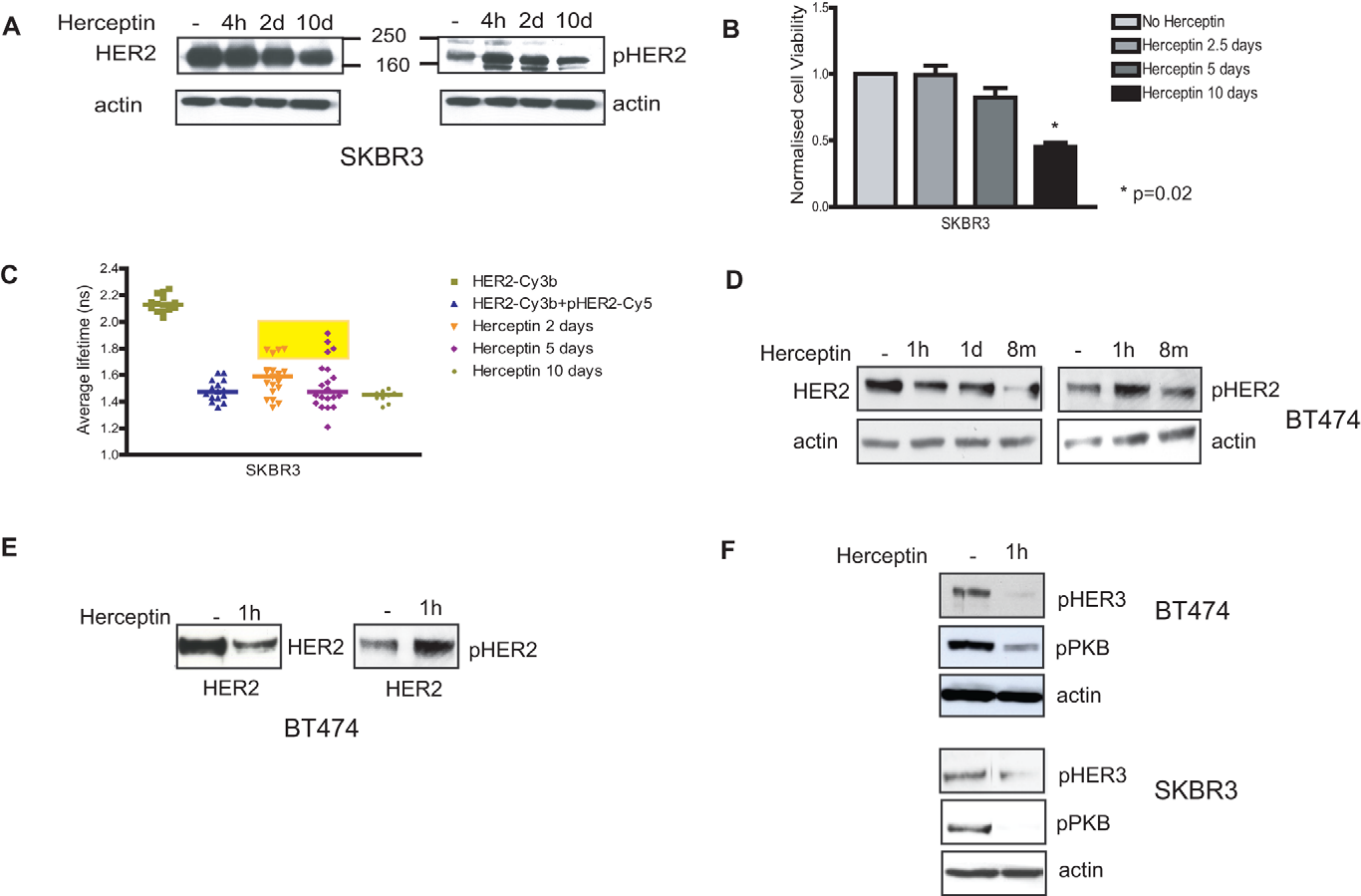
Herceptin down-regulates HER2 receptors and decreases HER3 phosphorylation, but it does not decrease HER2 phosphorylation. (A) SKBR3 cells were lysed for Western blot analysis after pre-treatment with 40 µg/ml Herceptin for a period of 4 h, 2 d, or 10 d. Equal amounts of protein were loaded in each lane, and two SDS-PAGE gels were run. Membranes were blotted with appropriate antibodies for total and phosphorylated HER2 and actin. (B) SKBR3 cells were grown in 24-well plates and left to grow for at least 24 h before being treated with 40 µg/ml Herceptin for different durations, as illustrated. The viable cells were counted in a cell viability analyzer using trypan blue to stain the dead cells. (C) SKBR3 cells were incubated with either donor alone (HER2-Cy3b) or donor and acceptor (HER2-Cy3b+pHER2-Cy5) to assess HER2 phosphorylation by FRET after pre-treatment with 40 µg/ml Herceptin for different durations, as illustrated. (D) BT474 cells were lysed for Western blot analysis after pre-treatment with 40 µg/ml Herceptin for a period of 1 h, 1 d, or 8 mo with replacement every week. Equal amounts of protein were loaded in each lane. Membranes were blotted with antibodies for total and phosphorylated HER2 and actin. (E) BT474 cells were immunoprecipitated with an antibody for cytoplasmic anti-HER2 (1∶100). Following the immunoprecipitation the cell lysate was loaded on an SDS gel. The membrane was probed with antibodies for total or phosphorylated HER2. (F) BT474 and SKBR3 cells were lysed after pre-treatment with 40 µg/ml Herceptin for 1 h. Equal amounts were loaded on an SDS gel, and membranes were probed for phosphorylated HER3, phosphorylated PKB, and actin.

Using the classical biochemical methods, we confirmed that Herceptin down-regulated HER2 receptors in sensitive SKBR3 cells after 10 d of treatment (Figure 1A). We then assessed the effect of Herceptin on HER2 phosphorylation. Herceptin did not decrease nor abolish HER2 phosphorylation (Figure 1A). Paradoxically, it increased HER2 phosphorylation in SKBR3 cells. However, despite an increase in HER2 phosphorylation, there was a decrease in cell viability in SKBR3 cells after 10 d of Herceptin treatment compared to untreated cells (*p = *0.02) (Figure 1B). Since a Western blot is unable to assess HER2 phosphorylation in individual cells or assess heterogeneity between cells, we proceeded to use FRET to assess HER2 phosphorylation in individual cells.

Using an established method to assess HER2 phosphorylation by FRET [16],[17], we conjugated an anti-HER2 antibody to a Cy3b fluorophore (HER2-Cy3b) and an anti-phospho-HER2 antibody to Cy5 (pHER2-Cy5) to assess HER2 phosphorylation in fixed SKBR3 cells with or without 40 µg/ml Herceptin (see Materials and Methods). The median donor lifetime of Cy3b was 2.15 ns (Figure 1C). HER2 phosphorylation would bring the donor and acceptor fluorophores into close proximity, resulting in a decrease of donor lifetime. We first monitored the basal phosphorylation in SKBR3 cells (without Herceptin treatment) and found a decrease in the average lifetime of HER2-Cy3b when pHER2-Cy5 was present (from 2.15 ns to 1.4 ns) (Figure 1C). Following Herceptin treatment, there was considerable heterogeneity between the cells, with suppression of HER2 phosphorylation in a few cells, although the phosphorylation of HER2 was maintained in the majority of cells (Figure 1C). After 10 d of Herceptin treatment, the remaining treated cells still had persistent HER2 phosphorylation (Figure 1C), and this represented approximately 50% of the cell number compared to untreated cells (*p = *0.02) (Figure 1B). Herceptin has been shown to target ALDH-positive stem cells in HER2-overexpressing breast cancer cells [18],[19]. We proceeded to show that after 6 d of Herceptin treatment, there was a decreased proportion of cells that were ALDH positive compared to untreated cells, correlated with a decrease in HER2 receptors (Figure S1).

As control, the effect of Herceptin on HER2 phosphorylation in the normal breast epithelial cell line MCF12F was also assessed. Even though acute Herceptin treatment could not inhibit HER2 phosphorylation in SKBR3 cells, it was able to decrease HER2 phosphorylation (shown by increase of lifetime) in MCF12F cells (Figure S2A). The inability of Herceptin to inhibit HER2 phosphorylation in SKBR3 cells was not due to the degradation of Herceptin (Figure S2B).

We also observed similar results in another HER2-overexpressing cell line, BT474 (Figure 1D). These cells were also sensitive to Herceptin treatment after several days of treatment, with decreased cell viability compared to control (Figure S3A, upper panel). As in SKBR3 cells, acute Herceptin exposure did not decrease HER2 phosphorylation in these cells (Figure 1D). HER2 phosphorylation increased in BT474 cells after 1 h of Herceptin treatment (Figure 1D). After treating these cells with Herceptin for 8 mo (with replacement of Herceptin every week), the cells became resistant to 40 µg/ml Herceptin (Figure S3A, lower panel). Herceptin was able to decrease but not eliminate ALDH-positive stem cells in long-term Herceptin-treated BT474 cells compared to untreated cells (Figure S3B). The decrease in ALDH-positive cells correlated with down-regulation of HER2 receptors. However, HER2 phosphorylation and cell viability remained in these resistant cells treated for a prolonged period with Herceptin (Figure 1D).

### The Effects of Acute Herceptin Treatment on HER3 and PKB Phosphorylation

We found that the down-regulation of HER2 receptors was detectable after 1 h of Herceptin treatment in BT474 cells, and was associated with an increase in HER2 phosphorylation (Figure 1E). Lee-Hoeflich et al. [20] showed that knockdown of HER2 receptors but not EGFR caused a significant decrease of HER3 phosphorylation in HER2-positive breast cell lines. We investigated whether this occurred in our experiments. After 1 h of Herceptin treatment in SKBR3 and BT474 cells, there was a decrease in HER3 phosphorylation correlating with a down-regulation of HER2 receptors (Figure 1F). HER2-overexpressing cells have been shown to constitutively suppress PTEN activity with increased PKB activity, and it has been shown that acute Herceptin exposure decreased PKB phosphorylation through PTEN activation [13]. We found that after 1 h of Herceptin treatment, there was a decrease in PKB phosphorylation, and this correlated with a decrease in HER3 phosphorylation in both SKBR3 and BT474 cells (Figure 1F). Thus, acute Herceptin treatment down-regulated HER2 receptors, resulting in a decrease of HER3 phosphorylation and PKB phosphorylation.

### HER2 Phosphorylation Is Maintained by Its Dimerisation with Other HER Receptors through Ligand Release

**Figure 2:**
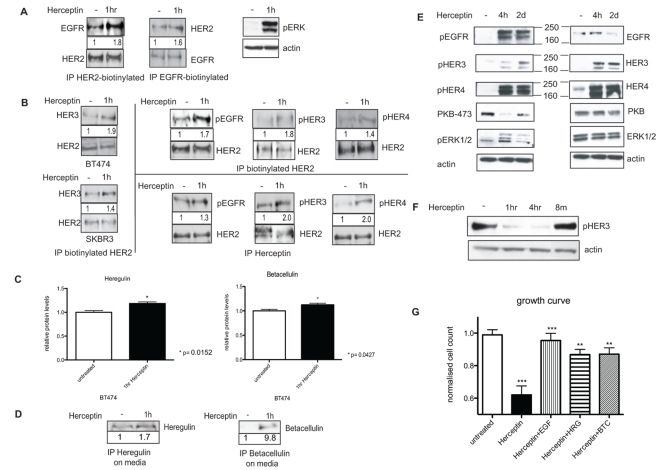
Herceptin induces the activation of HER receptors and their dimerisation with HER2 as a result of an up-regulation and the release of HER ligands. (A) In the left two panels, BT474 cells were immunoprecipitated (IP) with either intracellular anti-EGFR or anti-HER2 antibody after 1 h of 40 µg/ml Herceptin treatment. Following the immunoprecipitation, the cell lysate was loaded unto an SDS gel, and a Western blot analysis was performed. The membrane was probed with either anti-EGFR or anti-HER2 antibody, as illustrated. In the right panel, BT474 cells treated for 1 h with 40 µg/ml Herceptin were lysed, and equal amounts were loaded on a gel. Membrane was probed for phosphorylated ERK and actin. (B) In the left upper and lower panels, BT474 and SKBR3 cells were immunoprecipitated with biotinylated anti-HER2 antibody after the cells were treated for 1 h with 40 µg/ml Herceptin. Following the immunoprecipitation, equal amounts of the cell lysates were loaded unto an SDS gel, a Western blot analysis was performed, and the membrane was probed with anti-HER3 antibody. In the top right three panels, BT474 cells were immunoprecipitated with biotinylated anti-HER2 antibody after 1 h of Herceptin treatment. Western blot analysis was done using antibodies that recognised pEGFR, pHER3, or pHER4. In the lower right three panels, BT474 cells were treated with 40 µg/ml Herceptin for 1 h before the lysate was immunoprecipitated using the anti-HER2 antibody Herceptin. After immunoprecipitation, lysate was loaded onto an SDS gel. A Western blot analysis was performed, and the membrane was probed with anti-pEGFR, anti-pHER3, or anti-pHER4 antibodies. (C) BT474 cells were treated for 1 h with 40 µg/ml Herceptin, and the cells were lysed using Hepes buffer and homogenised before analysing the levels of heregulin and betacellulin by ELISA. (D) In the left panel, serum-free medium of untreated and Herceptin-treated SKBR3 cells was immunoprecipitated for heregulin. A Western blot analysis was then performed, and the membrane was probed for heregulin. In the right panel the same technique was used to look at betacellulin levels in the medium of SKBR3 cells. (E) SKBR3 cells were lysed for Western blot analysis after pre-treatment with 40 µg/ml Herceptin. An equal amount of protein was loaded in each lane. Four parallel SDS-PAGE gels were run, and the membranes were cut in different parts according to molecular weight to analyse the total and phosphorylation levels of HER receptors, PKB, ERK, and actin using appropriate antibodies. (F) BT474 cells were lysed after pre-treatment with 40 µg/ml Herceptin for 1 h, 4 h, or 8 mo. Equal amounts were loaded on an SDS gel, and membranes were probed for phosphorylated HER3 and actin. (G) BT474 cells were treated for 5 d with either 40 µg/ml Herceptin alone or concurrent 40 µg/ml Herceptin treatment with exogenous 100 ng/ml EGF, betacellulin (BTC), or heregulin (HRG) stimulation. Cells were then counted using a cell counter to assess cell viability. For statistical analysis, untreated and Herceptin-treated samples were compared using the Mann-Whitney test. The Herceptin-treated samples were then compared with samples treated with Herceptin and concurrent growth factor stimulation.

The amplification of HER2 results in constitutive activation of HER2 in a human mammary epithelial cell system [21]. We found that although Herceptin down-regulated HER2 receptors, the remaining cells had persistent and increased HER2 phosphorylation in both SKBR3 and BT474 cells (Figure 1A and 1D). Since HER2 is the preferred dimerisation partner, we postulated that HER2 phosphorylation was maintained by the other HER receptors via their dimerisation with HER2. We proceeded to show, using the streptavidine-biotin immunoprecipitation method (see Materials and Methods), that acute Herceptin treatment increased EGFR/HER2 dimerisation in BT474 cells (Figure 2A, left two panels). This effect was specifically induced by Herceptin, since 1 h of IgG treatment did not increase EGFR/HER2 dimerisation (data not shown). There was also an increase in HER2/HER3 dimerisation in both SKBR3 and BT474 cells after 1 h of Herceptin treatment (Figure 2B, left upper and lower panels). Furthermore, there was increased HER2 dimerisation with the phosphorylated EGFR and HER3 receptors in BT474 cells treated with Herceptin (which was demonstrated using two immunoprecipitation methods; see Materials and Methods) (Figure 2B, middle two upper and lower panels). There was also increased HER2 dimerisation with the phosphorylated HER4 receptor (Figure 2B, right upper and lower panels). Because of a low level of HER4 expression in these cells, the quality of the Western blot was not optimal using the streptavidin-biotin immunoprecipitation method, despite repeated attempts (Figure 2B, right upper panels). However, the quality of the blot was better using the immunoprecipitation method with Herceptin (Figure 2D, right lower panels).

We postulated that the increased dimerisation of EGFR, HER3, and HER4 with HER2 was due to activation by their respective ligands. We proceeded to assess the levels of endogenous ligands, using heregulin (ligand for HER3 and HER4) and betacellulin (ligand for EGFR and HER4) as examples. Herceptin-treated cells were lysed, and endogenous ligand levels were detected using ELISA. We found that Herceptin induced a statistically significant up-regulation of heregulin and betacellulin (*p = *0.0152 and *p = *0.0286, respectively) after 1 h of Herceptin treatment compared to untreated cells in both SKBR3 and BT474 cells (data on BT474 cells are shown in Figure 2C). There was also increased secretion of these ligands in the conditioned medium of these cells (Figure 2D). Thus, Herceptin increased the dimerisation of EGFR, HER3, and HER4 with HER2 as a result of activation by their ligands.

### Herceptin Induces Phosphorylation of EGFR and HER4 As Well As Reactivation of HER3 during Prolonged Treatment

We showed that Herceptin induced an up-regulation of HER ligands, including betacellulin and heregulin (Figure 2C and 2D). This resulted in an increased phosphorylation of EGFR and HER4 (Figure 2E) and an increase in their dimerisation with HER2 (Figure 2A and 2B). Herceptin, however, decreased HER3 phosphorylation initially after 1 h of Herceptin treatment (Figure 1F). We postulated that the increased heregulin release (Figure 2C and 2D) with Herceptin treatment would have an effect on HER3 phosphorylation. We showed that with prolonged Herceptin treatment, HER3 phosphorylation was reactivated in SKBR3 cells (Figure 2E). Reactivation of HER3 phosphorylation also occurred in BT474 cells that became resistant to Herceptin (Figure 2F). The total expression of HER3 and HER4 increased in SKBR3 cells treated with Herceptin, but the total EGFR expression decreased (Figure 2E). Thus, Herceptin induced ligand activation of EGFR and HER4 as well as reactivation of HER3 phosphorylation during prolonged Herceptin treatment.

### The Discordant Effects of Herceptin on ERK and PKB Pathways

We analysed the effects of Herceptin on the downstream signalling pathways in HER2-positive breast cancer cells. It was found that the effects of acute Herceptin treatment on phosphorylation of PKB and ERK1/2 were not concordant (Figures 1F, 2A, and 2E), in contrast to acute tyrosine kinase inhibitor (TKI) treatment, which decreased both PKB and ERK phosphorylation [17]. Acute Herceptin exposure increased ERK phosphorylation (Figure 2A and 2E) but decreased PKB phosphorylation in BT474 and SKBR3 cells (Figure 1F).

Acute Herceptin exposure increased EGFR/HER2 and HER2/HER4 dimerisation, correlating with an increase in ERK phosphorylation (Figure 2A and 2E). In contrast, acute Herceptin treatment decreased PKB phosphorylation (Figures 1F and 2E); this decrease has been shown to be due to activation of PTEN [13], correlating with a decrease in HER3 phosphorylation (Figure 1F). With prolonged Herceptin treatment, reactivation of PKB and HER3 occurred (Figure 2E). The increased ERK phosphorylation was transient (Figure 2A and 2E), mimicking the effect of exogenous ligand stimulation.

Therefore, Herceptin treatment decreased PKB phosphorylation because of a decrease in HER3 phosphorylation induced by HER2 down-regulation. However, 1 h of Herceptin treatment increased ERK phosphorylation as a result of ligand-dependent EGFR and HER4 activation.

### Exogenous HER Ligands Render Sensitive BT474 Cells Resistant to Herceptin Treatment

To further show that HER ligands play a role in the acquired resistance to Herceptin, we stimulated BT474 cells with 100 ng/ml EGF, heregulin, or betacellulin while they were treated with 40 µg/ml Herceptin. After 5 d, we assessed their cell viability. For Herceptin treatment without exogenous ligands, there was a decreased cell viability of BT474 cells, which was statistically significant (*p<*0.001) (Figure 2G). However, when Herceptin treatment was given in BT474 cells with concurrent stimulation of exogenous HER ligands, the decrease in cell viability was reversed. The reverse in cell viability in these conditions was statistically significant compared to Herceptin treatment alone (*p = *0.0001 for EGF, *p = *0.002 for heregulin, *p = *0.003 for betacellulin, respectively, compared to Herceptin alone) (Figure 2G).

### Herceptin Treatment Induces the ADAM17-Mediated Up-Regulation of HER Ligands

**Figure 3:**
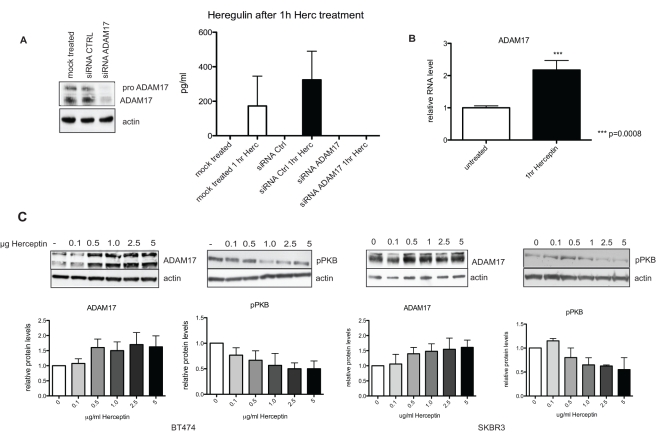
Up-regulation of heregulin is mediated by ADAM17, and acute Herceptin treatment induces an increase in mRNA and protein levels of ADAM17. (A) SKBR3 cells were transfected with siRNA against ADAM17 or a control sequence. The knockdown was validated by Western blot for ADAM17 and actin (left panel). Knockdown cells were then treated with 40 µg/ml Herceptin for 1 h and lysed in Hepes buffer for heregulin detection using ELISA (right panel). (B) SKBR3 cells were treated with 40 µg/ml Herceptin for 1 h, and ADAM17 mRNA levels were studied by QPCR. The Mann-Whitney test was performed to determine statistical significance of the up-regulation of ADAM17 mRNA after Herceptin treatment. (C) BT474 cells (left two panels) and SKBR3 cells (right two panels) were treated with an increasing dose of Herceptin over a period of 1 h. Cell lysate was loaded onto an SDS gel, and a Western blot analysis was performed. The membrane was probed with anti-ADAM17 and anti-pPKB antibodies. Quantification of three separate experiments is depicted in graphs for pPKB and ADAM17; representative blots are shown above the graphs.

We investigated the role of ADAM proteases since they mediate shedding of pro-HER ligands including HB-EGF, epiregulin, heregulin, and betacellulin [22]. As ADAM17 is one of the most important ADAM proteases for HER ligands, we studied the role of this ADAM protease in Herceptin treatment. SKBR3 cells were transfected with small interfering RNA (siRNA) against ADAM17, and the knockdown was validated by Western blot. There was a decrease in both pro and active forms of ADAM17 in transfected cells compared to the control (Figure 3A, left panels). We also showed that heregulin production increased in response to acute Herceptin exposure in cells transiently transfected with control siRNA, but this production was inhibited by siRNA against ADAM17 (Figure 3A, right panel). We also assessed the effect of Herceptin on the expression of ADAM17 protease. We demonstrated that after treating the cells with Herceptin for 1 h, ADAM17 protease mRNA was increased by 2.2-fold (*n* = 4, *p = *0.0008) (Figure 3B). To assess whether an increase in mRNA production of ADAM17 was translated to protein, we proceeded to assess ADAM17 protein level in response to Herceptin treatment. We showed that Herceptin increased the protein levels of ADAM17 in a dose-dependent manner after 1 h of treatment in both BT474 (Figure 3C, left two panels) and SKBR3 (Figure 3C, right two panels) cells. Furthermore, the increase of ADAM17 protease was shown to correlate with the suppression of PKB phosphorylation by Herceptin (Figure 3C). As a control, we treated MCF12F cells with Herceptin for comparison. Herceptin did not cause significant pPKB inhibition nor affect ERK phosphorylation in these cells after 1 h of treatment (Figure S2C, right panels). In addition, Herceptin did not induce a significant increase in ADAM17 level nor HER ligand levels (heregulin and betacellulin) in the conditioned medium of the normal epithelial breast cell line MCF12F treated with Herceptin (Figure S2C).

In summary, we showed that ADAM17 is involved in the up-regulation of heregulin in response to Herceptin treatment. We found increased levels of mRNA and protein levels of ADAM17 protease in response to acute Herceptin exposure in HER2-positive cells but not in normal epithelial MCF12F cells. The up-regulation of ADAM17 was shown to correlate with the suppression of PKB phosphorylation in HER2-overexpressing cells.

### Decrease in PKB Phosphorylation Initiates the Negative Feedback Loop of ADAM17-Mediated HER Ligand Release

We observed earlier that the up-regulation of ADAM17 correlated with the suppression of PKB phosphorylation by Herceptin treatment, suggesting the existence of a negative PKB feedback loop involving ADAM17 in acute Herceptin treatment. We hypothesized that if there was a negative PKB feedback loop, a PKB inhibitor should initiate the same response as Herceptin treatment, inducing an up-regulation of HER ligands and ADAM17 levels. To assess the role of a PKB feedback loop induced by Herceptin treatment, we treated BT474 cells with a PKB/Akt inhibitor (Akt inhibitor VIII, Akti-1/2), which can decrease PKB phosphorylation via a mechanism different from that of Herceptin.

**Figure 4:**
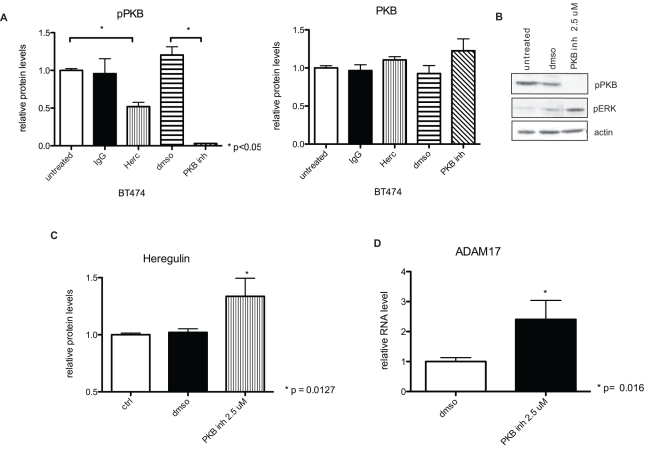
Inhibiting PKB phosphorylation by a PKB inhibitor induces up-regulation of heregulin and ADAM17. (A) BT474 cells were treated for 1 h with 40 µg/ml Herceptin (Herc), 2.5 µM PKB inhibitor (PKB inh), 40 µg/ml IgG control, or DMSO control at 37°C. After 1 h, cells were lysed and analysed for total and phosphorylated PKB levels using MSD multiplex kits. (B) BT474 cell lysates were loaded on an SDS gel, and the membranes were probed for phosphorylated ERK, PKB, and actin. (C) BT474 cells pre-treated with PKB inhibitor were lysed in Hepes buffer and homogenised before analysing protein levels of heregulin using ELISA. (D) BT474 cells were treated with PKB inhibitor for 1 h, and ADAM17 mRNA levels were studied by QPCR. Statistical significance was determined using the Mann-Whitney test.

Using the quantitative Meso Scale Discovery (MSD) method (see Materials and Methods), we showed that the PKB inhibitor decreased PKB phosphorylation after 1 h of treatment, which was statistically significant in comparison to DMSO control treatment (*p<*0.0001) (Figure 4A, left panel). Herceptin also decreased PKB phosphorylation in comparison with untreated cells (*p = *0.03 compared to untreated) (Figure 4A, left panel). Neither PKB inhibitor nor Herceptin decreased total PKB levels in comparison to control cells treated with IgG or DMSO (Figure 4A, right panel).

We also assessed the effects of the PKB inhibitor on PKB and ERK phosphorylation in BT474 cells using Western blot. As expected, 1 h of treatment with 2.5 µM PKB inhibitor, but not DMSO, decreased PKB phosphorylation. However, it also increased ERK phosphorylation (Figure 4B), just like acute Herceptin treatment (Figure 2A and 2E). More importantly, the decrease in PKB phosphorylation by the PKB inhibitor was also associated with an increase in heregulin production (*p = *0.012) (Figure 4C) and an up-regulation of ADAM17 mRNA levels (*p = *0.016) (Figure 4D). Thus, the decrease in PKB phosphorylation by a PKB inhibitor initiated the same feedback loop as that seen in Herceptin treatment, which reduces PKB phosphorylation via a different mechanism.

### Inhibition of ADAM17 Abrogates the PKB Negative Feedback Loop, Resulting in Suppression of HER2 Phosphorylation

In order to further prove the existence of a PKB negative feedback loop involving ADAM17 during Herceptin treatment, we needed to demonstrate that we can abrogate the loop and suppress HER2 phosphorylation by inhibiting ADAM17.

**Figure 5:**
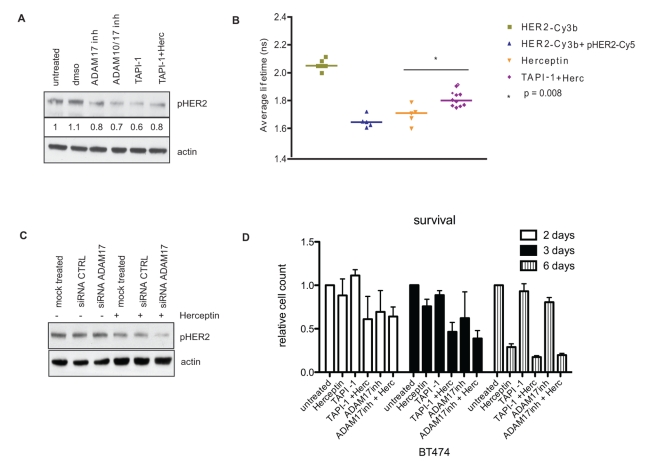
Combination of Herceptin with ADAM inhibitors decreases HER2 phosphorylation and is additive in cell viability inhibition. (A) SKBR3 cells were treated with 100 µM ADAM17 inhibitor (inh), 100 µM ADAM10/17 inhibitor, 100 µM ADAM inhibitor TAPI-1, or a combination of TAPI-1 and 40 µg/ml Herceptin (Herc) for 1 h. Cells were then lysed, and equal amounts of protein were loaded on an SDS gel. Membrane was probed for phosphorylated HER2 and actin. (B) FRET experiments to assess HER2 phosphorylation in SKBR3 cells. The cells were treated with 40 µg/ml Herceptin for 1 h with or without TAPI-1. The medians of the lifetimes were compared with the basal condition using the Mann-Whitney test. (C) SKBR3 cells were transfected with siRNA against ADAM17 or a control sequence. Three days after transfection, cells were treated with 40 µg/ml Hercpetin for 1 h. Lysates were loaded on an SDS gel. The membrane was probed for pHER2 and actin. (D) BT474 cells were grown in 24-well plates and left to grow for at least 24 h before being treated for different durations with 40 µg/ml Herceptin, 10 µM TAPI-1, 10 µM ADAM17 inhibitor, or a combination of ADAM inhibitors with Herceptin, as illustrated. The viable cells were counted using a cell counter.

HER2-overexpressing cells express autocrine ligands, including heregulin, resulting in HER2 activation and basal phosphorylation. We showed that 1 h of treatment with TAPI-1 (an ADAM17 and metalloprotease inhibitor) or with specific ADAM17 inhibitor and ADAM10/17 inhibitor was able to inhibit basal HER2 phosphorylation in SKBR3 cells (Figure 5A). Whereas acute Herceptin exposure increased HER2 phosphorylation in SKBR3 cells (Figure 1A), combination treatment with Herceptin and TAPI-1 decreased HER2 phosphorylation (Figure 5A). We also investigated the effect of the combination of Herceptin and TAPI-1 in individual SKBR3 cells using FRET. There was a basal phosphorylation of HER2 in SKBR3 cells, as shown by a decrease in the average lifetime of HER2-Cy3b with pHER2-Cy5 from about 2.05 ns to 1.6 ns (Figure 5B). Acute Herceptin treatment did not decrease HER2 phosphorylation (Figure 1A and 1C), but with concurrent TAPI-1 inhibitor treatment there was suppression of HER2 phosphorylation (an increase in the average lifetime) (*p = *0.008) (Figure 5B).

To further prove the role of ADAM17 in the negative feedback loop, we transiently transfected SKBR3 cells with siRNA against ADAM17. We showed that Herceptin was unable to decrease HER2 phoshorylation in control cells. However, in cells that were transfected with specific siRNA against ADAM17, HER2 phosphorylation was decreased after Herceptin treatment (Figure 5C).

We proceeded to assess the effect of various ADAM17 inhibitors on cell viability with or without concurrent Herceptin treatment. We hypothesized that the combination of Herceptin with ADAM17 inhibitors would exert greater inhibition than Herceptin alone. We showed that combination of Herceptin with either TAPI-1 or specific ADAM17 inhibitor exerted greater inhibition of cell viability in BT474 cells after 2, 3, or 6 d of treatment (Figure 5D).

Thus, our data prove that inhibition of ADAM17 is able to abrogate the feedback loop that maintains HER2 phospshorylation during Herceptin treatment.

### A panHER Inhibitor Abrogates the PKB Negative Feedback Loop, and the Combined Treatment of a panHER Inhibitor with Herceptin Is Synergistic in Inhibiting Xenograft Tumour Growth

We demonstrated earlier that ADAM17 inhibitors were able to abrogate the PKB negative feedback loop and inhibit HER2 phosphorylation during Herceptin treatment. Since an up-regulation of ADAM17 and HER ligands resulted in activation of all HER receptors, we hypothesized that a panHER inhibitor should also be able to reverse the effect of the PKB negative feedback loop induced by Herceptin treatment.

**Figure 6:**
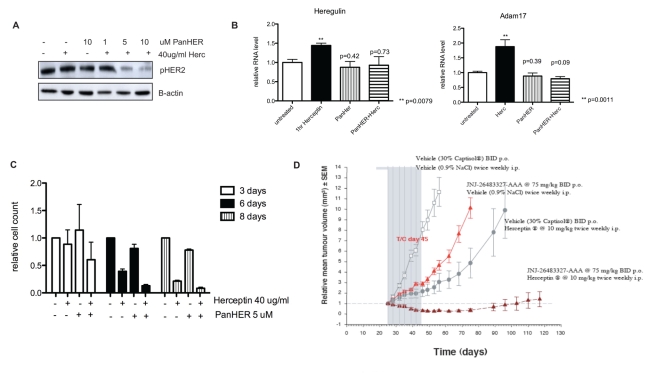
Combination of Herceptin with a panHER inhibitor abrogates the PKB feedback loop and is synergistic in inhibition of xenograft tumour growth. (A) SKBR3 cells were treated for 1 h with 40 µg/ml Herceptin (Herc) alone, 10 µM panHER inhibitor JNJ-26483327 alone, or Herceptin with 1, 5, or 10 µM JNJ-26483327. Cell lysate was loaded onto an SDS gel, and a Western blot analysis was performed. Membranes were blotted with antibodies recognising pHER2 and β-actin. (B) BT474 cells are treated with 40 µg/ml Herceptin, 5 µM JNJ-26483327, or a combination of both drugs for 1 h. ADAM17 mRNA and heregulin mRNA levels were studied by QPCR. *p-*values were calculated using the Mann-Whitney test comparing treated samples against untreated samples. (C) BT474 cells were grown in a 24-well plate and left to grow for at least 24 h before being treated for different durations with 40 µg/ml Herceptin, 5 µM JNJ-26483327, or a combination of these drugs, as illustrated. The viable cells were counted using a cell counter. (D) BT474 xenografts were treated with an empty vehicle, JNJ-26483327 (75 mg/kg twice a day orally [BID p.o.]), Herceptin (10 mg/kg twice weekly intraperitonealy [i.p.]), or a combination of JNJ-26483327 with Herceptin treatment for 21 d and were observed for a period of 120 d.

We investigated whether a panHER inhibitor, which inhibits the activation of all HER receptors, could decrease HER2 phosphorylation and be synergistic in tumour growth inhibition with Herceptin treatment. JNJ-26483327 is a potent multi-kinase inhibitor against EGFR (half-maximal inhibitory concentration [IC_50_] = 9.6 nM), HER2 (IC_50_ = 18 nM), and HER4 (IC_50_ = 40.3 nM) [23] (Figure S4A). It is also known as a panHER inhibitor since its IC_50_ against these receptors is comparable with that of other panHER inhibitors [24]. We found that 1 h of treatment with either 40 µg/ml Herceptin or 10 µM JNJ-26483327 was not able to decrease HER2 phosphorylation in SKBR3 cells (Figure 6A). However, the combination of Herceptin treatment with either 5 µM or 10 µM JNJ-26483327 for 1 h was able to decrease HER2 phosphorylation in these cells (Figure 6A). Furthermore, whereas Herceptin treatment alone increased ADAM17 (*p = *0.011) and heregulin (*p = *0.008) mRNA levels, neither JNJ-26483327 treatment nor the combined treatment of Herceptin with JNJ-26483327 increased their levels compared to the untreated cells (Figure 6B). Therefore, the combined treatment of a panHER inhibitor with Herceptin could abrogate the PKB feedback loop involving ADAM17 and heregulin. We also showed that the combination of Herceptin and panHER inhibitor JNJ-26483327 exerted greater inhibition of cell viability after 3, 6, or 8 d of treatment compared to Herceptin or JNJ-26483327 alone (Figure 6C). We investigated whether a panHER inhibitor in combination with an ADAM17 inhibitor without Herceptin treatment could exert similar synergistic effect. However, JNJ-26483327 with TAPI-1 exerted less cell viability inhibition than Herceptin alone in both SKBR3 and BT474 cells (Figure S4C). This may be because neither JNJ-26483327 alone nor JNJ-26483327 with TAPI-1 could decrease pHER3 and pAKT after 1 h of treatment, in contrast to Herceptin or Herceptin with JNJ-26483327 (Figure S4B).

To test the in vivo relevance of the interaction between a panHER inhibitor and Herceptin, BT474 xenografts were treated with an empty vehicle, Herceptin alone, the panHER inhibitor JNJ-26483327 alone, or the combination of drugs for 21 d (Figure 6D). As seen in Figure 6D, Herceptin alone or the panHER inhibitor alone could only delay xenograft tumour growth compared to vehicle treatment, but the combination of the two drugs caused an almost complete inhibition of tumour growth in these HER2-positive xenografts.

In summary, Herceptin in combination with a panHER inhibitor was able to decrease HER2 phosphorylation and abrogated the up-regulation of ADAM17 and heregulin in response to Herceptin treatment. Whereas either a panHER inhibitor or Herceptin treatment alone delayed BT474 xenograft tumour growth, the combination treatment was synergistic in tumour inhibition.

## Discussion

It was previously thought that Herceptin inhibits HER2 receptor tyrosine kinase activity, but recent studies have shown that this is not the case [14],[15]. The mechanisms whereby Herceptin fails to decrease HER2 phosphorylation remain unclear. Our results confirmed that Herceptin did not decrease HER2 phosphorylation although it down-regulated HER2 receptors in HER2-positive SKBR3 and BT474 breast cell lines. We showed that HER2 phosphorylation was maintained and increased by the ligand-induced activation of EGFR, HER3, and HER4 receptors, which preferentially dimerise with HER2. This is consistent with reports that Herceptin does not prevent the dimerisation of HER2 with other receptors [25].

It was previously demonstrated that primitive mammary stem cells are enriched in vitro in non-adhering spherical colonies called mammospheres [26]. These cells have stem-cell-like properties, with the ability to undergo multi-lineage differentiation [26]. The proportion of these stem cells in normal mammary epithelial cells is increased by HER2 overexpression, as demonstrated by in vitro mammosphere assays and the expression of the stem cell marker ALDH [18]. One of the clinical benefits of Herceptin is thought to be its ability to target the cancer stem cell population in HER2-amplified tumours [19]. We confirmed in our study that Herceptin decreased ALDH-positive stem cells after a prolonged treatment, in correlation with a decrease in HER2 receptors. However, there was significant heterogeneity in the inhibition of HER2 phosphorylation by Herceptin between different cells, especially during the first week of treatment. Thus, Herceptin was able to decrease HER2-mediated signalling in some of these cells, resulting in decreased HER2 receptors and phosphorylation, but Herceptin monotherapy could not eliminate all the stem cells. The surviving cells had decreased HER2 receptors and fewer ALDH-positive cells compared to untreated cells but had maintained HER2 phosphorylation via activation of other HER receptors as a result of ADAM17-mediated ligand release.

HER3 is kinase-defective, and its phosphorylation depends on other HER receptors. The effects of Herceptin on HER3 phosphorylation have been controversial. Yakes et al. [27] showed that 1 h of Herceptin treatment induced a transient increase of pHER3 in BT474 cells, whereas Junttila et al. [15] reported a decrease of pHER3 with acute Herceptin treatment. We showed that acute Herceptin treatment initially decreased HER3 phosphorylation. This decrease is thought to be due to HER2 down-regulation, since loss of HER2 induced by siRNA decreased pHER3 levels in HER2-positive breast cancer cells [20]. It is possible that the difference in observed pHER3 is due to several factors that are competing to affect pHER3 levels. The dominant effect of acute Herceptin treatment is a decrease in HER2 levels, but this is in competition with the increased HER3 phosphorylation as a result of increased ligand production induced by Herceptin treatment. This would account for the subsequent reactivation of HER3 with prolonged Herceptin treatment. Furthermore, variable cell lines and experimental conditions, as well as different treatment durations and doses of Herceptin used by different investigators, may account for differences in pHER3 levels seen [15],[27].

We found that Herceptin had a discordant effect on PKB and ERK signalling. HER2-overexpressing cells have been shown to activate Src with constitutively suppressed PTEN activity and increased PKB activity [13],[28]. Herceptin increased PTEN membrane localisation and phosphatase activity by reducing PTEN tyrosine phosphorylation via Src inhibition, leading to a decreased PKB phosphorylation [13]. The decrease in PKB activity is not due to PI3K inhibition, since there was no decreased PI3K activity after Herceptin treatment [13]. However, acute Herceptin activates ERK1/2 pathways, correlating with an increase in EGFR and HER4 dimerisation with HER2. Since EGFR, HER3, and HER4 have binding sites for Shc and Grb2 [29], their ligand-dependent activation would account for increased activation of ERK by acute Herceptin treatment. The sudden increase in ERK phosphorylation induced by Herceptin treatment is very much similar to MAPK/ERK activation in cells stimulated with exogenous HER ligands [30]. This observation supports our data that Herceptin induces the up-regulation of HER ligands through ADAM proteases.

**Figure 7:**
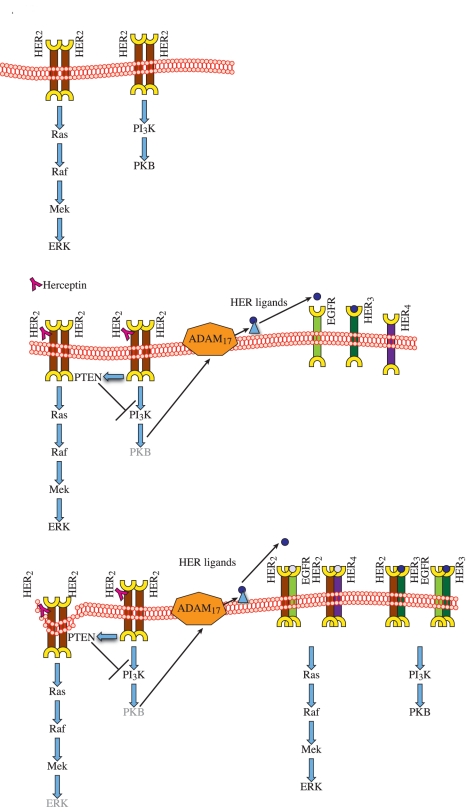
The proposed model of acquired resistance to Herceptin in HER2-overexpressing breast cancer. In the top diagram, HER2 overexpression (both homodimers and heterodimers) leads to constitutive activation of EGFR and HER2 with MAPK activation [21]. These cells have also been shown to have activated Src with constitutively suppressed PTEN activity and increased PKB activity [13],[28]. In the middle diagram, Herceptin increases PTEN membrane localisation and PTEN activation in HER2-overexpressing cells, resulting in suppression of PKB phosphorylation [13]. This inhibition induces the PKB negative feedback loop, triggering the production and release of HER ligands through ADAM17 protease. In the bottom diagram, Herceptin down-regulates HER2 receptors, but the up-regulation of HER ligands causes activation of EGFR, HER3, and HER4 and their dimerisation with HER2, resulting in the maintenance of HER2 phosphorylation, with increased activation of PKB and MAPK pathways.

Sergina et al. [31] showed that TKI treatment failed to suppress HER3 phosphorylation for a sustained duration because of a PKB-mediated feedback loop. However, they did not link the PKB-mediated feedback loop with the HER ligands and ADAM proteases. We have previously shown that reactivation of HER3 and PKB in response to TKI is due to HER ligand release [16]. We have now shown that acute Herceptin treatment decreases the phosphorylation of HER3 and PKB, which in turn induces the activation of a feedback loop involving HER ligands and ADAM proteases. We postulated that Herceptin treatment initiated a PKB-mediated negative feedback loop. If such a negative loop exists, we predicted that an inhibitor that decreases PKB phosphorylation should also induce the up-regulation of HER ligands and ADAM17 protease. Indeed, we demonstrated that a PKB inhibitor, which decreases PKB phosphorylation via a mechanism different from that of Herceptin, could also initiate the same feedback loop induced by Herceptin treatment. Thus, it is a Herceptin-induced decrease in PKB phosphorylation that results in the activation of a feedback loop involving ADAM proteases and HER ligands. This PKB feedback loop activates the other HER receptors and maintains HER2 phosphorylation, which is a key to acquired resistance to Herceptin (Figure 7).

There have been several reports of positive and negative feedback loops linking the complex cross talk of MAPK and PI3K signalling pathways with scaffolding protein Grb2-associated binders 1 and 2 (Gab1 and Gab2) [30],[32]. mTOR inhibition has also been shown to lead to MAPK activation through a PI3K-dependent feedback loop in human cancer [33]. The exact mechanism of how these feedback loops link to each other is likely to be very complex. It is likely that FoxO proteins, which are downstream targets of PKB, are central players in this PKB feedback loop [34]. The phosphorylation of FoxO transcription factors by PKB promotes the cytoplasmic sequestration of these transcription factors, including FoxO3a [34]. It is likely that FoxO transcriptional factors can modify Akt/PKB phosphorylation indirectly by modifying the expression of kinases or phosphatases [35]. It is also possible that FoxO proteins regulate the transcription of ADAM17, since PKB inhibition increases mRNA production of ADAM17. The interactions of FoxO transcriptional factors with ADAM proteases and phosphatase, as well as how they affect the phosphorylation and dephosphorylation of PKB and HER receptors, are likely to be very complicated. The mechanisms are currently being investigated in our lab.

Our xenograft experiment showed that neither panHER inhibitor nor Herceptin treatment alone was adequate to control tumour growth in a HER2-oncogene-driven tumour. In HER2-positive breast cancer, the underlying problem is HER2 overexpression, which results in increased HER2-related signalling. Herceptin is able to down-regulate HER2 receptors, whereas TKI-like lapatinib induces HER2 accumulation at the cell surface [14]. We have shown that a panHER inhibitor with TAPI-1 (ADAM17 inhibitor) resulted in less inhibition of cell viability than Herceptin alone in both SKBR3 and BT474 cells. This confirms that TKI treatment without Herceptin is not as effective as treatment with either Herceptin or Herceptin with TKI in HER2-positive breast cancer cells. However, the combination of Herceptin with a panHER inhibitor, which inhibits the activation of all HER receptors, was able to abrogate the feedback loop during Herceptin treatment and was synergistic in tumour inhibition in a HER2-positive BT474 xenograft model. Our data would support the rationale of combining a panHER inhibitor with Herceptin treatment in patients with HER2-positive breast cancer. Our results would also explain why pertuzumab and Herceptin are synergistic in tumour inhibition in breast cancer cells and xenograft models [20]. Furthermore, it would account for the effectiveness of a combination treatment of pertuzumab with Herceptin in some patients whose disease progressed while on Herceptin alone [36].

Our results demonstrate why it is inadequate to consider individual HER receptors alone for anti-HER therapies as these receptors are intrinsically linked together with a close network of feedback loop(s). We have demonstrated that a PKB feedback loop is activated upon Herceptin treatment, resulting in the activation of all HER receptors and maintenance of HER2 phosphorylation. We have not attempted to look at all the positive and negative feedback loops linking MAPK and PI3K [30],[32]. It is likely that more feedback loops are involved in the acquired resistance to Herceptin. Future research should identify the exact candidates, other than ADAM17 and HER ligands, involved in the feedback loops. Such candidates are best identified through a systems biology approach, which may help to further dissect the mechanisms of acquired resistance to Herceptin in HER2-overexpressing tumours and to improve the survival for patients with this type of tumour.

## Materials and Methods

### Materials and Cell Lines

SKBR3 and BT474 cells were obtained from cell services at Cancer Research UK (Lincoln's Inn Fields laboratory). The human cell lines BT474 and SKBR3 are HER2-overexpressing breast cancer cell lines. SKBR3 cells were cultured in Dulbecco's Modified Eagle Medium (DMEM) supplemented with 10% fetal bovine serum and the antibiotic penicillin-streptomycin. BT474 cells were cultured in RMPI supplemented with 10% fetal bovine serum and the antibiotics penicillin-streptomycin. For these cells, 10 µg/ml insulin was added to the medium when cells were split or medium was refreshed. Normal breast epithelial MCF12F cells were purchased from the ATCC and cultured in a 1∶1 mixture of DMEM and Ham's F12 medium supplemented with 20 ng/ml EGF, 100 ng/ml cholera toxin, 0.001 mg/ml insulin, 500 ng/ml hydrocortisone, and 5% horse serum.

Anti-HER2 (recognising the intracellular residues), anti-phospho-HER2 (Tyr1221/1222), anti-phospho-HER3 (Tyr1289), anti-HER4 (recognising the intracellular residues near the carboxyl-terminus of human HER4), and anti-phospho HER4 (Tyr1284) antibodies were obtained from Cell Signalling Technology. The polyclonal anti-phospho-EGFR (Thr992) was obtained from Invitrogen. F4-IgG1 mouse monoclonal antibody (against the EGFR cytoplasmic domain) was obtained from the monoclonal antibody laboratory of Cancer Research UK (Lincoln's Inn Fields laboratory). Antibodies recognising PKB, phospho-PKB (Ser473), p44/42 MAP kinase (Erk1/Erk2), and phospho-Erk1/Erk2 (Thr202/Tyr204) were from Cell Signalling Technology. Anti-ADAM17 was purchased from Abcam. The secondary antibodies, goat anti-mouse IgG and goat anti-rabbit IgG, were purchased from GE Healthcare. The mono-conjugated fluorophores Cy3B and Cy5 were from GE Healthcare. PKB inhibitor (Akt inhibitor VIII, isozyme-selective, Akti-1/2) was obtained from Calbiochem. Herceptin was initially a gift from Roche, but subsequent supply was obtained from the pharmacy department of Oxford Radcliffe Hospitals, National Health Services Trust. Human IgG control was purchased from R&D Systems. EGF, heregulin, and betacellulin were purchased from Sigma Aldrich. ADAM and metalloprotease inhibitor (TAPI-1) was purchased from Calbiochem. Incyte kindly provided ADAM17 inhibitor INCB4298 and ADAM10/17 inhibitor INCB3619. Janssen (Johnson & Johnson) kindly provided panHER inhibitor JNJ-26483327.

### Western Blotting

For Western blotting confluent six-well plates of cells were placed on ice and washed with PBS. Cells were scraped off the plates and incubated for 10 min in lysis buffer (10 mM EDTA, 20 mM Tris [pH 7.5], 150 mM NaCl, 10 mM Na_2_P_2_O_7_, and 100 mM NaF with 1% Triton and protease inhibitor cocktail [Roche]). Samples were centrifuged at 4°C to remove the insoluble cell pellets, and a protein assay was performed to check protein quantity. Equal amounts of protein sample were prepared in 4× SDS with 10% beta-mercaptoethanol and boiled for 10 min at 95°C. Then samples were loaded on a NuPage 4%–12% gel (Invitrogen) and run at 130 V. The proteins were semi-dry-transferred to a membrane for 2 h at 12 V. The membrane was blocked in 3% BSA in PBS-Tween (0.2%) for a minimum of 1 h. Then the blot was incubated with primary antibody in the same solution for 3 h at room temperature. The membrane was washed four times with 1% milk in PBS-Tween (0.2%) before secondary antibody was added in 5% milk in PBS-Tween (0.2%). The membrane was incubated at room temperature for 1 h before it was washed four times again with 1% milk in PBS-Tween (0.2%). Antibodies were visualised with an enhanced chemiluminescent (ECL) system (GE Healthcare).

### Immunoprecipitation

BT474 and SKBR3 cells were grown to near confluency before they were lysed as described above. The cell lysate was centrifuged for 10 min at maximum speed before transferring the supernatant to a new reaction vial. A protein assay was performed to check protein quantity, and equal amounts of protein were used for immunoprecipitation. In order to look at the interaction between HER2 and other HER receptors after Herceptin treatment, we needed a technique that would specifically pull down our protein of interest. We could not use magnetic protein G beads because they would bind Herceptin as well as our receptor-specific antibody (data not shown). Therefore, streptavidin-coated magnetic beads (Bio-Nobile) were absorbed with biotin-conjugated HER antibodies (1∶100) (conjugated using kit from Innova Biosciences) for 1 h, as illustrated in the figures. After the bead-antibody complex was washed with PBS-Tween (0.2%), it was incubated with the supernatant for at least 1 h. After that, the complex was thoroughly washed with PBS-Tween and transferred to a new reaction vial. Wash buffer was taken off and 50 µl of 4× SDS with 10% beta-mercaptoethanol was added to the beads. Samples were boiled for 10 min at 95°C to elude protein off the beads. Twenty microlitres was loaded per lane in a SDS gel for Western blot analysis, as described above. For further confirmation, we also used protein G beads labelled with Herceptin (anti-HER2) to pull down HER2, and looked at the levels of phosphorylated HER receptors.

### Cell Viability Experiments

SKBR3 and BT474 cells were grown in 24-well plates after seeding approximately 20,000 cells per well. The cells were grown for at least 24 h before treatment with 40 µg/ml Herceptin, 5 µM JNJ-26483327, 10 µM TAPI-1, 10 µM ADAM17 inhibitor, or a combination of these drugs for different durations, as illustrated in the figures. For the exogenous ligand experiments, 100 ng/ml EGF, heregulin, or betacellulin was added to the cells in addition to Herceptin (40 µg/ml) for a total of 5 d in BT474 cells. On the day of the experiment, the cells were trypsinized and diluted with PBS. The viable cells were counted using a cell counter.

### Transient Transfection

For all transient transfections, the cells were plated in 10-cm^2^ dishes (50% confluent) in medium and given the opportunity to settle overnight. The next day, medium was replaced by 7 ml of fresh normal medium. Transfection mix containing 10 nM siRNA in 300 µl of OptiMEM (Invitrogen) was incubated with 15 µl of NeoFX (Ambion) in 300 µl of OptiMEM for 15 min at room temperature. This mix was added drop-wise to the cells, and cells were placed back into the incubator. After 24 h, transfected cells were re-plated into six-well plates for further experiments. Validated siRNAs were obtained from Ambion.

### Real-Time Quantitative PCR Analysis

Cells treated with Herceptin (40 µg/ml), PKB inhibitor (2.5 µM), or panHER inhibitor JNJ-26483327 (5 µM) were analysed for mRNA levels of ADAM17 and heregulin. RNA was purified from cells using the Aurum Total RNA mini kit (Bio-Rad) according to manufacturer's instructions. RNA purity and quantity were determined using Nanodrop (Nanodrop Technologies). For synthesis of the cDNA, a high-capacity cDNA reverse transcription kit was used (Qiagen).

Quantitative PCR (QPCR) reactions were performed using the following primers, together with FAM-labelled probes from the Universal ProbeLibrary (Roche) or Sybergeen: Beta-actin primers, 5′-ATTGGCAATGAGCGGTTC-3′ and 5′-GGATGCCACAGGACTCCAT-3′, and universal probe 11; heregulin-β1 primers, 5′- CTTGTGGTCGGCATCATGT-3′ and 5′-CAGCTTTTTCCGCTGTTTCT-′3, and probe 49; ADAM17 primers, 5′-CCTTTCTGCGAGAGGGAAC-3′ and 5′- CACCTTGCAGGAGTTGTCAG-3′ and probe 78.

cDNA samples were assayed in triplicate using a detection system (Chromo4; GRI), and gene expression levels for each individual sample were normalised relative to Beta-actin. Levels of mRNA in untreated samples were set to 1.

### Quantification of HER Ligand Levels by Immunoprecipitation in Medium

We used immunoprecipitation to look at the levels of ligands (betacellulin and heregulin) in the media of untreated or Herceptin-treated cells. Cells were treated with Herceptin in serum-free medium for 1 h. The medium was then collected and incubated with magnetic protein G beads (Invitrogen) linked to antibodies for heregulin or betacellulin for at least 1 h. The samples were prepared for Western blot analysis as described above.

### Quantification of Ligand Levels by ELISA

Cells treated with Herceptin (40 µg/ml) or PKB inhibitor (2.5 µM) were analysed for HER ligands. Levels of the HER ligands betacellulin and heregulin were measured in cell lysate using ELISA (R&D Systems). Cells were lysed in 20 mM Hepes buffer containing 1.5 mM EDTA and protease inhibitor (Roche). Cells were then scraped off and homogenised by putting the lysate through a syringe with needle. After spinning the lysate down, the supernatant was used for ELISA analysis.

To detect heregulin or betacellulin, the R&D Systems ELISA kits were used as the protocol prescribes. Briefly, the ELISA plate was coated overnight with coating buffer. After blocking the plate for 2 h, 100-µl samples and standards were loaded and the plate was kept at room temperature for 2 h. Samples were washed and incubated with detection antibody for 2 h, then washed and incubated with streptavidin labelled with horseradish peroxidase for 20 min. With the use of a substrate solution that reacts with horseradish peroxidase, the levels of ligands could be detected. After 20 min, the reaction was stopped using stop solution, and absorption was measured using an ELISA reader.

### Quantification of Phosphorylated PKB and PKB Levels by MSD

BT474 cells were plated on a six-well plate and left to settle overnight. The next day, the cells were treated for 1 h with 40 µg/ml Herceptin or 2.5 µM PKB inhibitor at 37°C. After 1 h, the cells were lysed in a lysis buffer provided by MSD. The assay was then performed following manufacturer's protocol. In brief, MSD provided the plates pre-coated with pPKB (Ser473) and PKB spots. Multiple spots could be placed in one well of a 96-well plate, making it possible to look at several proteins in one sample. The plates provided were blocked for 1 h with 3% BSA at room temperature. The plates were then washed three times with wash buffer before 25 µl (20 µg) of sample was loaded onto the plates (in duplicate). After incubation for 2 h at room temperature, the plates were washed again, and 25 µl of detection antibody in 1% BSA was added. After 1 h of incubation, 150 µl of read buffer was added, and the plate was analyzed using a SECTOR imager (MSD).

### Förster Resonance Energy Transfer

FRET experiments were performed as described previously [16],[17]. In short, 30,000 cells were seeded onto cover slips and left to attach overnight. The next day, the cells were treated with 40 µg/ml Herceptin or the ADAM inhibitor TAPI-1 or the combination of these drugs (for different durations as illustrated) in the medium at 37°C. The medium was washed off with PBS, and the cells were fixed with 4% paraformaldehyde (Pierce) in PBS for 10 min at room temperature. The cells were then permeabilized with 0.2% Triton X-100 (T8532, Sigma-Aldrich) in PBS for 5 min at room temperature before being treated for 10 min with 1 mg/mg sodium borohydrate (Sigma-Aldrich) in PBS to quench the background fluorescence. The cells were washed twice with PBS between steps. Following the above steps, the cells were treated for 1 h with in 1% BSA (Sigma-Aldrich) in PBS at room temperature to block unspecific binding of the antibodies. After that, antibodies conjugated to the fluorescent dyes Cy3b or Cy5 were added to the samples sequentially starting with the Cy3b-conjugated antibody. Cells were washed twice with PBS and twice with sterile water, after which they were mounted onto a microscope slide using Fluoromount-G (Southern Biotech). All images were taken using a Zeiss Plan-APOCHROMAT 6100/1.4 NA phase three-oil objective. Images were recorded at a modulation frequency of 80 MHz. The donor (Cy3b) was excited using the 514-nm line of an argon/krypton laser, and the resultant fluorescence was separated using a combination of dichroic beam splitter (Q565 LP, Chroma Technology) and narrow band emitter filter (BP 610/75, Lys and Optik).

### ALDH Assay

To detect ALDH activity in cells, we used the ALDEFLUOR kit from Aldagen. The manufacturer's protocol was followed carefully. In brief, cells were trypsinized and, per treatment protocol, 1×10^6^ cells were taken up into 1 ml of assay buffer provided in the kit. This tube was the sample tube. To a separate control tube, 5 µl of DEAB solution was added. ALDEFLUOR substrate (5 µl) was added to the sample tube, and immediately after mixing, 500 µl of the sample solution was transferred to the control tube with DEAB. The procedure was repeated for all samples. Both control and sample tubes were then placed at 37°C for 45 min. After the cells were spun down for 5 min at 250 *g*, they were taken up in new assay buffer and analyzed using a FACS Cyan. Control samples were used to set a gate for ALDH positivity, after which the test sample was analysed.

### Statistical Analysis

The Mann-Whitney test was used to compare the medians of the protein levels of heregulin and betacellulin as well as mRNA levels of heregulin and ADAM17 between the untreated samples and those treated with drugs. For each figure, at least three experiments were done, and both technical and biological replicates were used in the calculation, using a confidence interval of 95%. Data were analysed against the untreated samples unless otherwise stated.

### Xenograft Experiment

#### Cell line

The human BT474 breast tumour cell line was derived from a 60-y-old female Caucasian patient, and supplied by OncoDesign. Cells were cultured at 37°C in a humidified atmosphere (5% CO_2_, 95% air), in DMEM supplemented with 2 mM L-glutamine, 50 mg/ml gentamicin, 1 mM sodium pyruvate, 1.5 g/l sodium bicarbonate, 4 mg/ml insulin, and 10% fetal bovine serum. Cells were maintained as monolayer cultures, being passaged once weekly at 1×10^7^ cells per T175 flask using the following procedure. Briefly, cells were washed with PBS, before the addition of trypsin-EDTA to the culture flasks. After detachment of cells the trypsin-EDTA was inactivated by addition of complete medium. The cell suspension was then transferred to a 50-ml Falcon tube and centrifuged for 3 min at 1,200 rpm. The medium was aspirated, with the cells being resuspended in an appropriate volume of complete medium. The cells were counted in a hemocytometer, and their viability was assessed by 0.25% trypan blue exclusion. An appropriate volume of cell suspension was then added to either a new T175 culture flask(s) or a roller bottle containing fresh medium. For a large scale-up growth of BT474 breast tumour cells, an appropriate number of triplet flasks were seeded with 20×10^6^ cells 1 wk prior to inoculation of mice. The medium was changed twice during this period, with the last change being the day prior to cell injection. Cells were collected as described above, with the exception that after centrifugation, the cells were resuspended in cold (4°C) serum-free medium.

#### Study design

Human BT474 breast tumour cells were injected directly into the inguinal region of the male NMRI Nude mice (1×10^7^ cells/200 ml/animal) on day 0. On day 25, when the tumour volume had reached an approximate average of 200 mm^3^, mice were randomized according to tumour volume, with ten mice per treatment group. Mice were then treated twice daily with either vehicle (30% Captisol) or vehicle containing JNJ-26483327 (75 mg/kg twice daily) by gavage (p.o.) for 21 d. Alternatively, mice were treated twice weekly with either vehicle (0.9% [w/v] NaCl) or a vehicle containing Herceptin (10 mg/kg) by intraperitoneal injection administered in a volume of 10 ml/kg body weight, for three cycles (i.e., 21 d). Another group received combined doses of both agents. Tumour size and body weights were measured twice weekly, with mice monitored daily for clinical signs of toxicity for the duration of the treatment. Clinical signs of toxicity included (but were not limited to) persistent anorexia or dehydration, morbidity, lethargy, hypothermia, and/or laboured respiration (according to the United Kingdom Coordinating Committee on Cancer Research guidelines for the welfare of animals in experimental neoplasia).

#### Data analysis

For each individual animal, body weight and tumour size (using the commonly accepted formula: tumour volume [mm^3^] = *a*×*b* ^2^/2, where *a* represents the length, and *b* the width of the tumour as determined by caliper measurements) were monitored twice weekly throughout the study. A sustained body weight loss greater than 15% of the initial body weight was considered as clinical toxicity, with the animal removed from the study and sacrificed. Time course of tumour growth was expressed as median values, or normalised to initial tumour volume on the day treatment started and expressed as mean ± standard error of the mean (eight animals per group). For pre-established tumours, relative tumour volumes were calculated for each mouse (treated tumour volume/tumour volume on day 0) and expressed as mean ± standard error of the mean for each treatment group. Twenty-four hours after their last treatment, animals were sacrificed, and tumours were excised and weighed. Anti-tumour effect of compound versus control was determined and represented by a bar chart of median values ±25/75 and 10/90 percentiles. Statistical significance was indicated by one-sided *p*-values from the Wilcoxon Mann-Whitney analysis (Wilcoxon rank sum test), and *p*<0.05 was considered as statistically significant. Treatment/control ratios were calculated based on final relative tumour volumes, using the National Cancer Institute criteria.

## Supporting Information

Figure S1 **ALDH activity after treatment with Herceptin.** SKBR3 cells were treated with 40 µg/ml Herceptin for 2, 4, or 6 d and were then analyzed for percentage of stem cells using the ALDH assay. The experiment was repeated three times, and the average percentage of cells positive for ALDH is depicted in a graph. FACS plots of untreated SKBR3 cells and SKBR3 cells treated with Herceptin for 6 d are depicted. On the left, the control samples are shown, where the ALDH reaction is blocked using DEAB. Negative cells are gated (R2). Using this gate on the right figures, the percentage of ALDH-negative cells can be found. Cells that fall outside of this gate are ALDH positive.
(1.31 MB TIF)

Figure S2 **Control experiments.** (A) In this experiment, MCF12F cells were incubated with either the donor alone (HER2-Cy3b) or donor and acceptor (HER2-Cy3b+pHER2-Cy5), to assess HER2 phosphorylation by FRET after being pre-treated with different durations of 40 µg/ml Herceptin as illustrated. (B) Western blot experiment using the medium from the SKBR3 cells treated with 40 µg/ml Herceptin as well as medium containing 40 mg/ml Herceptin that was kept in an incubator for up to 10 d. The medium was denatured with SDS-PAGE and boiled for 10 min, and 40 µl of 40 mg/ml Herceptin was loaded in each lane of the SDS-PAGE. The membrane was probed with monoclonal anti-human immunoglobulin antibody that recognises the Fc component of Herceptin. (C) MCF12F cells were treated with 10 µg/ml Herceptin for 1 h in serum-free medium. The medium was analysed for heregulin (left, top panel) and betacellulin (left, bottom panel) using immunoprecipitation. Cells were lysed and analysed by Western blot for ADAM17, pPKB, PKB, pERK, ERK, and actin (right panels).
(1.14 MB TIF)

Figure S3 **Chronic treatment with Herceptin induces its resistance and decreases ALDH activity in BT474 cells.** (A) BT474 cells were treated with 40 µg/ml Herceptin for 3 or 6 d. The cells were trypsinized and counted using a cell counter. In the middle panel, BT474 cells and BT474 cells cultured with 40 µg/ml Herceptin for over 8 mo (Herceptin-resistant BT474 cells) were treated for 6 d with 0, 20, or 40 µg/ml Herceptin. The cells were then trypsinized and counted using a cell counter. (B) Untreated BT474 cells and long-term Herceptin-treated BT474 cells (40 µg/ml Herceptin for over 8 mo) were analyzed using the ALDH assay. The average percentage of cells positive for ALDH is depicted in a graph.
(0.26 MB TIF)

Figure S4 **Kinase inhibition assay of JNJ-26483327 and its effect in combination with Herceptin or ADAM17 inhibitor TAPI-1.** (A) JNJ-26483327 (a multi-kinase inhibitor and also known as a panHER inhibitor) was tested against a panel of around 230 wild-type and mutant kinases at a concentration of 1 µM and an ATP concentration of 10 µM. Dose response tests were carried out for all those that showed greater than 50% inhibition. (B) SKBR3 cells were treated with 40 µg/ml Herceptin, 10 µM TAPI-1, a combination of TAPI-1 and Herceptin, 5 µM panHER inhibitor (JNJ-26483327), or a combination of the panHER inhibitor with TAPI-1. Western blot analysis was performed for pHER2, pHER3, pPKB, PKB, pERK, and ERK. (C) SKBR3 cells were treated with 40 µg/ml Herceptin, 10 µM TAPI-1, 5 µM JNJ-26483327, a combination of JNJ-26483327 with TAPI-1, or a combination of TAPI-1, JNJ-26483327, and Herceptin for a period of 6 d. The cells were then trypsinized and counted using a cell counter.
(1.00 MB TIF)

## Abbreviations

DMEM: Dulbecco's Modified Eagle Medium
FRET: Förster resonance energy transfer
IC_50_: half-maximal inhibitory concentration
MSD: Meso Scale Discovery
QPCR: quantitative PCR
siRNA: small interfering RNA
TKI: tyrosine kinase inhibitor
