# Correction: HER2 Phosphorylation Is Maintained by a PKB Negative Feedback Loop in Response to Anti-HER2 Herceptin in Breast Cancer

**DOI:** 10.1371/journal.pbio.1002414

**Published:** 2016-03-07

**Authors:** Merel Gijsen, Peter King, Tim Perera, Peter J. Parker, Adrian L. Harris, Banafshé Larijani, Anthony Kong

The authors would like to clarify that some of the blots previously depicted in Fig 1E and Fig 2B were for different experiments and were included in error.

The correct blots for Fig 1E and part of Fig 2B could not be located. These panels have therefore been removed from the revised versions included here after a careful assessment and investigation determined that the results for which the original figure panels were cited are supported elsewhere in this article, and that removal of these panels does not affect the conclusions of the paper.

The authors have provided new versions of Figs 1–5 to accommodate these changes. These revised figures now also clearly indicate blot splices that were not previously indicated or declared in Figs 1F and 2F, and replace incorrectly spliced blots with the un-spliced originals in Fig 4B. Those blots that are from the same experiment and share controls are now clearly indicated in the figure legends for Figs 1A, 2A, 2B, 2E, 3A and 5C. The authors have also corrected the inhibitor concentrations in the legend to Fig 5.

The text in the Results sections titled “The effects of acute Herceptin treatment on HER3 and PKB phosphorylation” and “The discordant effects of Herceptin on ERK and PKB pathways” has been edited to accommodate the removal of panel E in Fig 1, and the rearrangement of panel F in Fig 1, as explained above. The text in the Results section titled “HER2 phosphorylation is maintained by its dimerisation with other HER receptors through ligand release” has been edited to accommodate the removal of part of Fig 2B.

The corrected text, Figs 1–5 and their respective legends are included in this correction.

## The effects of acute Herceptin treatment on HER3 and PKB phosphorylation

We found that the downregulation of HER2 receptors was detectable after Herceptin treatment in SKBR3 and BT474 cells, and was associated with an increase in HER2 phosphorylation (Fig 1A and 1D). Lee-Hoeflich et al (2009) showed that knockdown of HER2 receptors but not EGFR caused a significant decrease of HER3 phosphorylation in HER2 positive breast cell lines [20]. We investigated whether this occurred in our experiments. After 1 hour of Herceptin treatment in SKBR3 and BT474 cells, there was a decrease in HER3 phosphorylation (Fig 1E and 1F), correlating with a downregulation of HER2 receptors (Fig 1A and 1D). HER2 over-expressing cells have been shown to constitutively suppress PTEN activity with increased PKB activity and it has been shown that acute Herceptin exposure decreased PKB phosphorylation through PTEN activation [13]. We found that after one hour of Herceptin treatment, there was a decrease in PKB phosphorylation and this correlated with a decrease in HER3 phosphorylation in both SKBR3 and BT474 cells (Fig 1E and 1F). Thus, acute Herceptin treatment downregulated HER2 receptors resulting in a decrease of HER3 phosphorylation and PKB phosphorylation.

## The discordant effects of Herceptin on ERK and PKB pathways

We analysed the effects of Herceptin on the downstream signalling pathways in HER2 positive breast cancer cells. It was found that the effect of acute Herceptin treatment on phosphorylation of PKB and ERK1/2 was not concordant (Figs 1E, 1F, 2A and 2E), in contrast to acute TKIs treatment which decreased both PKB and ERK phosphorylation [17]. Acute Herceptin exposure increased ERK phosphorylation (Fig 2A and 2E) but decreased PKB phosphorylation in BT474 and SKBR3 cells (Fig 1E and 1F).

Acute Herceptin exposure increased EGFR/HER2 and HER2/HER4 dimerisation, correlating with an increase in ERK phosphorylation (Fig 2A and 2E). In contrast, acute Herceptin treatment decreased PKB phosphorylation (Figs 1E, 1F and 2E), which has been shown to be due to activation of PTEN [13] correlating with a decrease in HER3 phosphorylation (Fig 1F). With prolonged Herceptin treatment, reactivation of PKB phosphorylation and HER3 occurred (Fig 2E). The increased ERK phosphorylation was transient (Fig 2A and 2E), mimicking the effect of exogenous ligand stimulation.

Therefore, Herceptin treatment decreased PKB phosphorylation due to a decrease in HER3 phosphorylation induced by HER2 downregulation. However, one hour of Herceptin treatment increased ERK phosphorylation as a result of ligand-dependent EGFR and HER4 activation.

## HER2 phosphorylation is maintained by its dimerisation with other HER receptors through ligand release

We found that although Herceptin downregulated HER2 receptors, the remaining cells had persistent and increased HER2 phosphorylation in both SKBR3 and BT474 cells (Fig 1A and 1D). Since HER2 is the preferred dimerisation partner, we postulated that HER2 phosphorylation was maintained by the other HER receptors via their dimerisation with HER2. We proceeded to show that acute Herceptin treatment increased EGFR/HER2 dimerisation in BT474 cells using the streptavidine-biotin immunoprecipitation method (see Methods) (Fig 2A, left two panels). This effect was specifically induced by Herceptin since one hour of IgG treatment did not increase EGFR/HER2 dimerisation (data not shown). There was also an increase in HER2/HER3 dimerisation in both SKBR3 and BT474 cells after one hour of Herceptin treatment (Fig 2B, left upper and lower panels). Furthermore, there was increased HER2 dimerisation with the phosphorylated EGFR and HER3 receptors in BT474 cells treated with Herceptin (which was demonstrated using two immunoprecipitation methods–see Methods) (Fig 2B, right upper two panels and middle lower two panels). There was also increased HER2 dimerisation with the phosphorylated HER4 receptor (Fig 2B, right lower panel). Due to a low level of HER4 expression in these cells, the quality of the western blot was not optimal using the streptavidin-biotin immunoprecipitation method despite repeated attempts. However, the quality of the blot was better using immunoprecipitation method with Herceptin (Fig 2B, right lower panel).

We postulated that the increased dimerisation of EGFR, HER3 and HER4 with HER2 was due to activation by their respective ligands. We proceeded to assess the levels of endogenous ligands, using heregulin (ligand for HER3 and HER4) and betacellulin (ligand for EGFR and HER4) as examples. Herceptin treated cells were lysed and endogenous ligand levels were detected using ELISA. We found that Herceptin induced a statistically significant upregulation of heregulin and betacellulin (p = 0.0152 and p = 0.0286 respectively) after 1 hour of Herceptin treatment compared to untreated cells in both SKBR3 and BT474 cells (data on BT474 cells are shown in Fig 2C). There was also increased secretion of these ligands in the conditioned medium in these cells (Fig 2D).

Thus, Herceptin increased the dimerisation of EGFR, HER3 and HER4 with HER2 as a result of the activation by their ligands.

**Fig 1:**
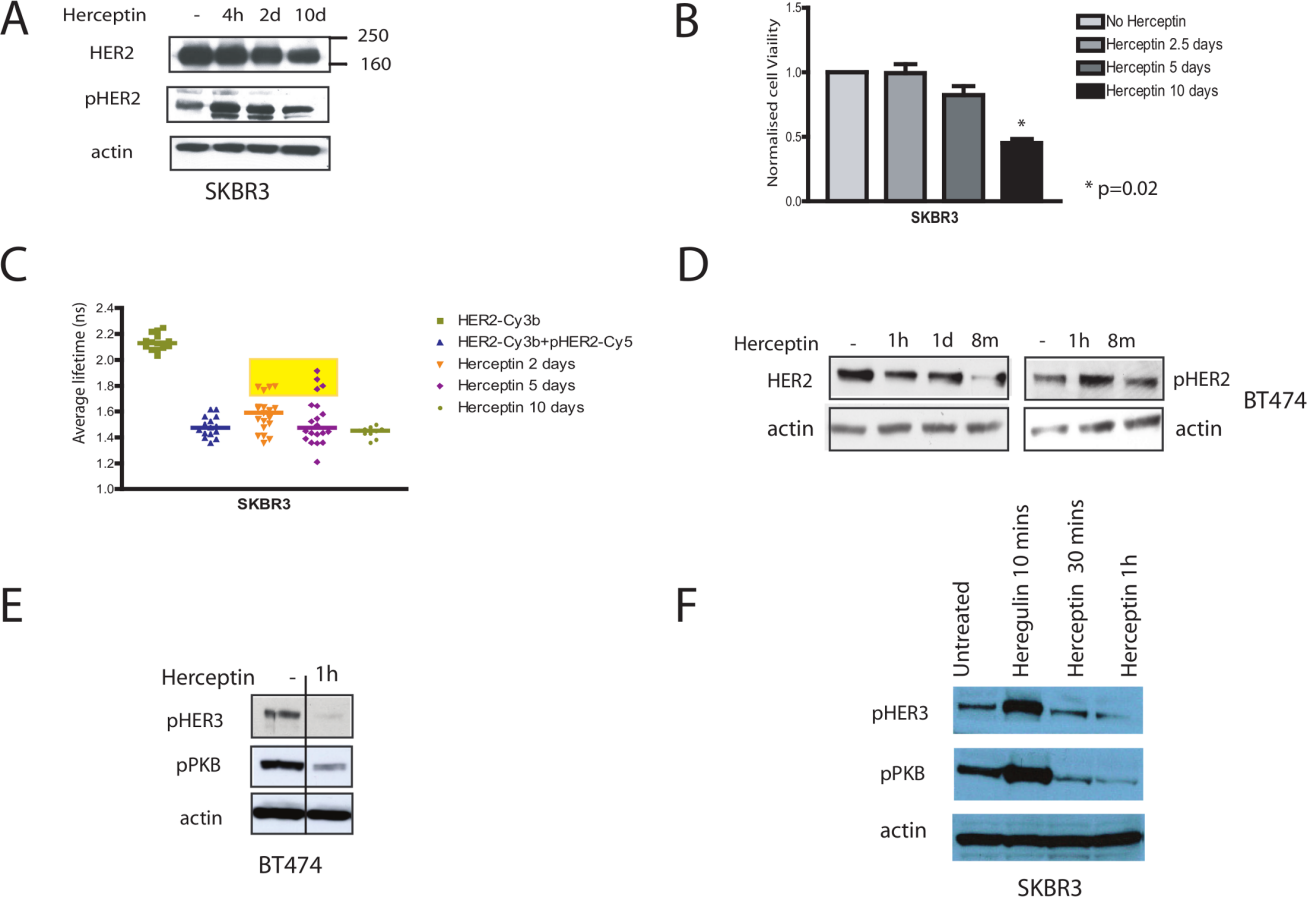
Herceptin down-regulates HER2 receptors and decreases HER3 phosphorylation, but it does not decrease HER2 phosphorylation. (A) SKBR3 cells were lysed for Western blot analysis after pre-treatment with 40 μg/ml Herceptin for a period of 4 h, 2 d, or 10 d. Equal amounts of protein were loaded in each lane, and multiple parallel SDS-PAGE gels were run. Membranes were blotted with appropriate antibodies to assess the indicated proteins (shown here and Fig 2E). Note that only one representative actin control is shown for this experiment. (B) SKBR3 cells were grown in 24-well plates and left to grow for at least 24 h before being treated with 40 μg/ml Herceptin for different durations, as illustrated. The viable cells were counted in a cell viability analyzer using trypan blue to stain the dead cells. (C) SKBR3 cells were incubated with either donor alone (HER2-Cy3b) or donor and acceptor (HER2-Cy3b+pHER2-Cy5) to assess HER2 phosphorylation by FRET after pre-treatment with 40 μg/ml Herceptin for different durations, as illustrated. (D) BT474 cells were lysed for Western blot analysis after pre-treatment with 40 μg/ml Herceptin for a period of 1 h, 1 d, or more than 8 months (m) with replacement every week. Equal amounts of protein were loaded in each lane. Membranes were blotted with antibodies for total and phosphorylated HER2 and actin. (E) BT474 cells were lysed for western blot analysis for the indicated proteins after 40 μg/ml Herceptin treatment for 1 hour. Note that other treatment conditions were assessed at the same time in other lanes of the same gel but they were cut from the membranes as they were irrelevant for these blots. The images were spliced from the same gel with a solid line is included to indicate the splice. (F) SKBR3 cells were lysed after 40 μg/ml Herceptin treatment or stimulation with heregulin 100ng/ml for the indicated durations. Equal amounts were loaded on an SDS gel, and membranes were probed for the indicated proteins.

**Fig 2:**
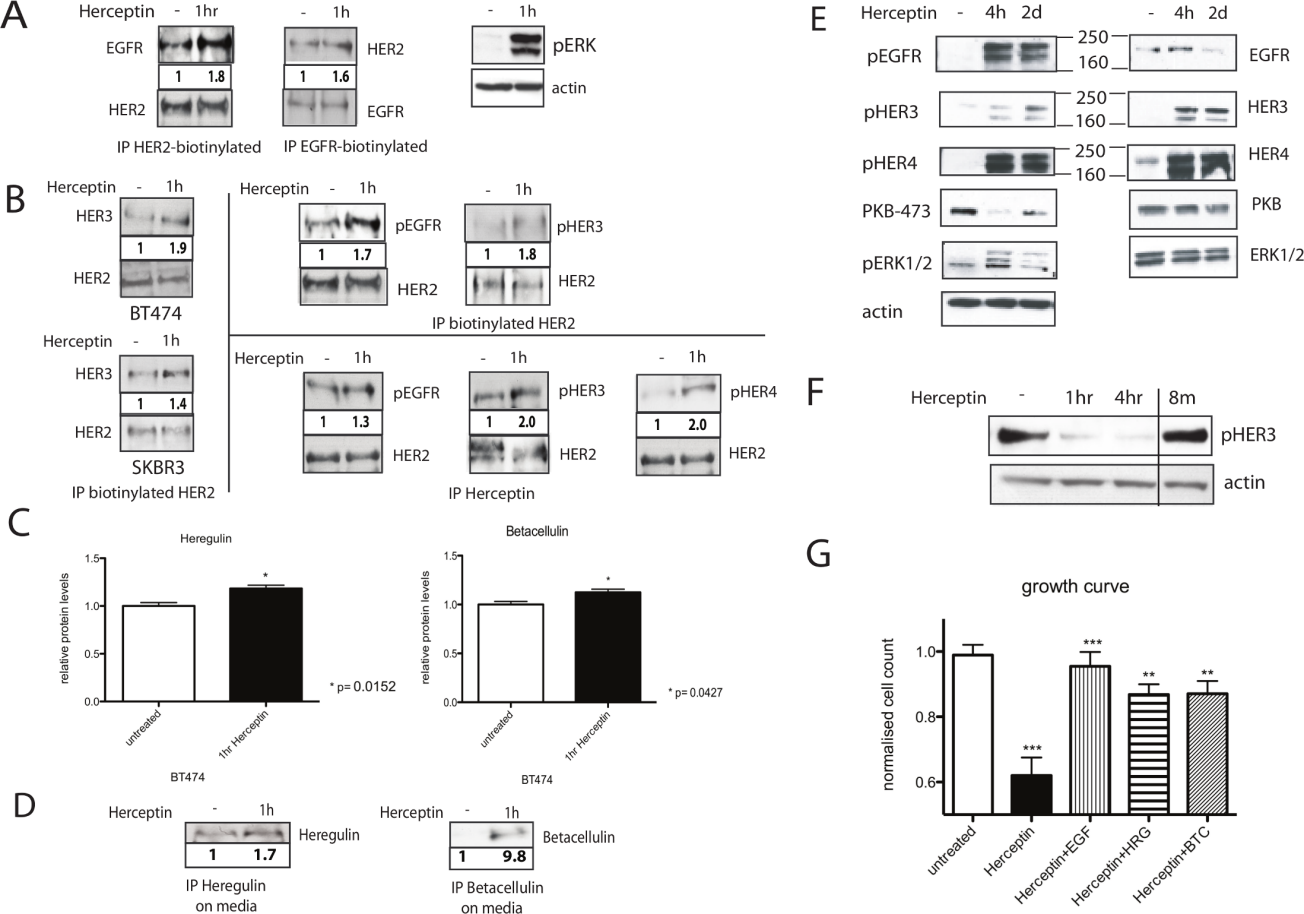
Herceptin induces the activation of HER receptors and their dimerisation with HER2 as a result of an up-regulation and the release of HER ligands. (A) In the left two panels, BT474 cells were immunoprecipitated (IP) with either intracellular anti-EGFR or anti-HER2 antibody after 1 h of 40 μg/ml Herceptin treatment. Following the immunoprecipitation, the cell lysate was loaded unto an SDS gel, and a Western blot analysis was performed. The membrane was probed with either anti-EGFR or anti-HER2 antibody, as illustrated. The cell lysate from those immunoprecipitated with anti-HER2 antibody, was also probed for pEGFR shown in Fig 2B upper second panel. In the right panel, BT474 cells treated for 1 h with 40 μg/ml Herceptin were lysed, and equal amounts were loaded on a gel. Membrane was probed for phosphorylated ERK and actin. (B) In the left upper and lower panels, BT474 and SKBR3 cells were immunoprecipitated with biotinylated anti-HER2 antibody after the cells were treated for 1 h with 40 μg/ml Herceptin. Following the immunoprecipitation, equal amounts of the cell lysates were loaded unto an SDS gel, a Western blot analysis was performed, and the membrane was probed with anti-HER3 antibody. In the top right two panels, BT474 cells were immunoprecipitated with biotinylated anti-HER2 antibody after 1 h of Herceptin treatment. Western blot analysis was done using antibodies that recognised pEGFR and pHER3. Note that pEGFR blot was from the same experiment using the same cell lysate immunoprecipitated with anti-HER2 antibody in Fig 2A as described above. In the lower right three panels, BT474 cells were treated with 40 μg/ml Herceptin for 1 h before the lysate was immunoprecipitated using the anti-HER2 antibody Herceptin. After immunoprecipitation, lysate was loaded onto an SDS gel. A Western blot analysis was performed, and the membrane was probed with anti-pEGFR, anti-pHER3, or anti-pHER4 antibodies. Note that the pEGFR and pHER4 blots were from the same experiment using the same cell lysate immunoprecipitated with Herceptin however the pHER3 blot was from a different experiment. (C) BT474 cells were treated for 1 h with 40 μg/ml Herceptin, and the cells were lysed using Hepes buffer and homogenised before analysing the levels of heregulin and betacellulin by ELISA. (D) In the left panel, serum-free medium of untreated and Herceptin-treated SKBR3 cells was immunoprecipitated for heregulin. A Western blot analysis was then performed, and the membrane was probed for heregulin. In the right panel the same technique was used to look at betacellulin levels in the medium of SKBR3 cells. (E) SKBR3 cells were lysed for Western blot analysis after pre-treatment with 40 μg/ml Herceptin as described in Fig 1A. An equal amount of protein was loaded in each lane. Multiple parallel SDS-PAGE gels were run, and the membranes were cut in different parts according to molecular weight to analyse the indicated proteins using the appropriate antibodies. Only one representative actin control was shown for this experiment. (F) BT474 cells were lysed after pre-treatment with 40 μg/ml Herceptin for 1 h, 4 h, or 8 m. Equal amounts were loaded on an SDS gel, and membranes were probed for phosphorylated HER3 and actin. Note that we also had treatment conditions related to Herceptin withdrawal on the same gel but these were cut from the gel for clarity and the images were spliced from the same gel. A solid line is now included to indicate the splice. (G) BT474 cells were treated for 5 d with either 40 μg/ml Herceptin alone or concurrent 40 μg/ml Herceptin treatment with exogenous 100 ng/ml EGF, betacellulin (BTC), or heregulin (HRG) stimulation. Cells were then counted using a cell counter to assess cell viability. For statistical analysis, untreated and Herceptin-treated samples were compared using the Mann-Whitney test. The Herceptin-treated samples were then compared with samples treated with Herceptin and concurrent growth factor stimulation.

**Fig 3:**
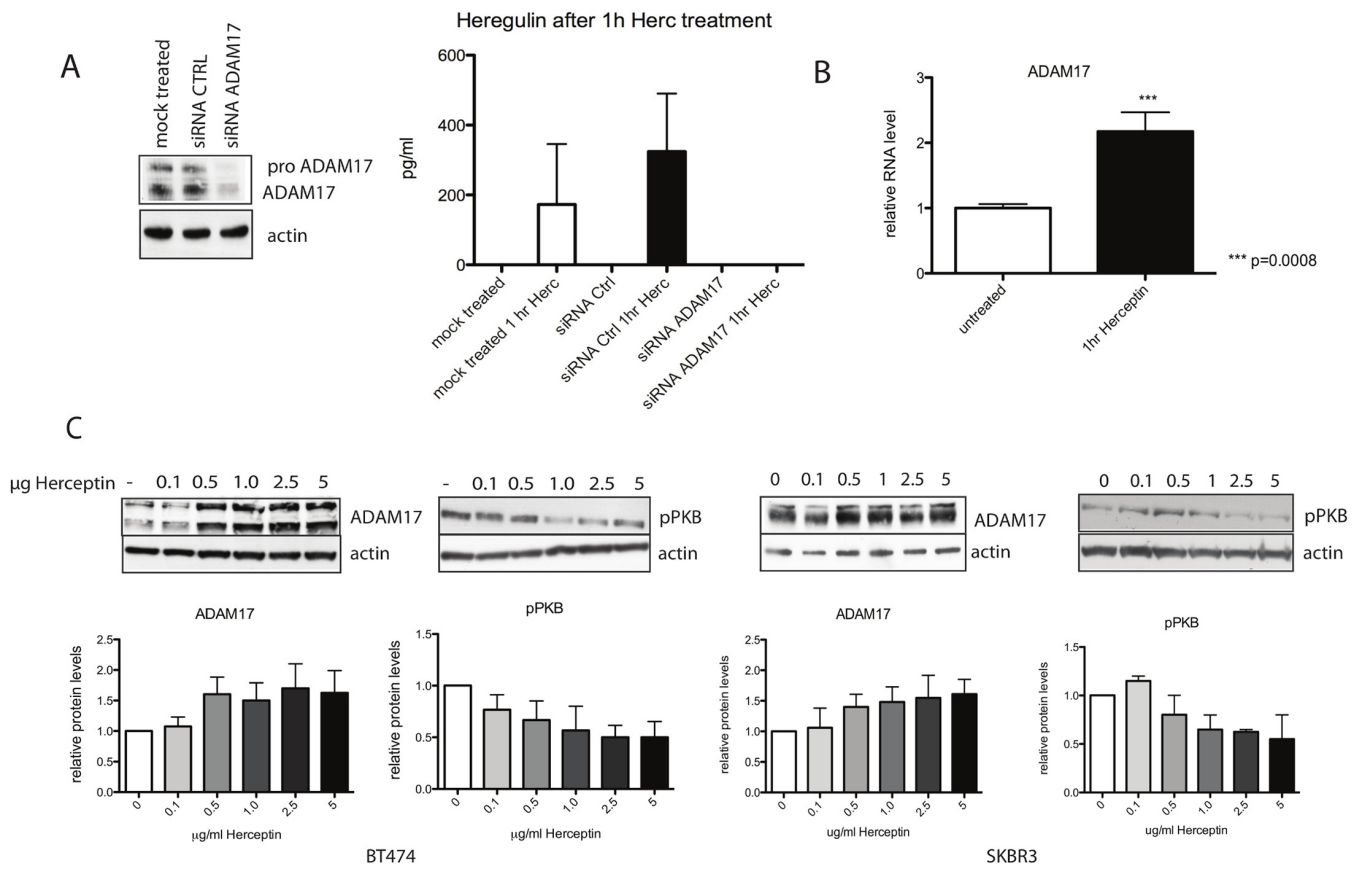
Up-regulation of heregulin is mediated by ADAM17, and acute Herceptin treatment induces an increase in mRNA and protein levels of ADAM17. (A) SKBR3 cells were transfected with siRNA against ADAM17 or a control sequence. Three days after transfection, cells were treated with 40 μg/ml Herceptin for 1 h. Lysates were loaded on an SDS gel. The membrane was probed for the indicated proteins (left panel and Fig 5C). The cells were also lysed in Hepes buffer for heregulin detection using ELISA (right panel). (B) SKBR3 cells were treated with 40 μg/ml Herceptin for 1 h, and ADAM17 mRNA levels were studied by QPCR. The Mann-Whitney test was performed to determine statistical significance of the up-regulation of ADAM17 mRNA after Herceptin treatment. (C) BT474 cells (left two panels) and SKBR3 cells (right two panels) were treated with an increasing dose of Herceptin over a period of 1 h. Cell lysate was loaded onto an SDS gel, and a Western blot analysis was performed. The membrane was probed with anti-ADAM17 and anti-pPKB antibodies. Quantification of three separate experiments is depicted in graphs for pPKB and ADAM17; representative blots are shown above the graphs.

**Fig 4:**
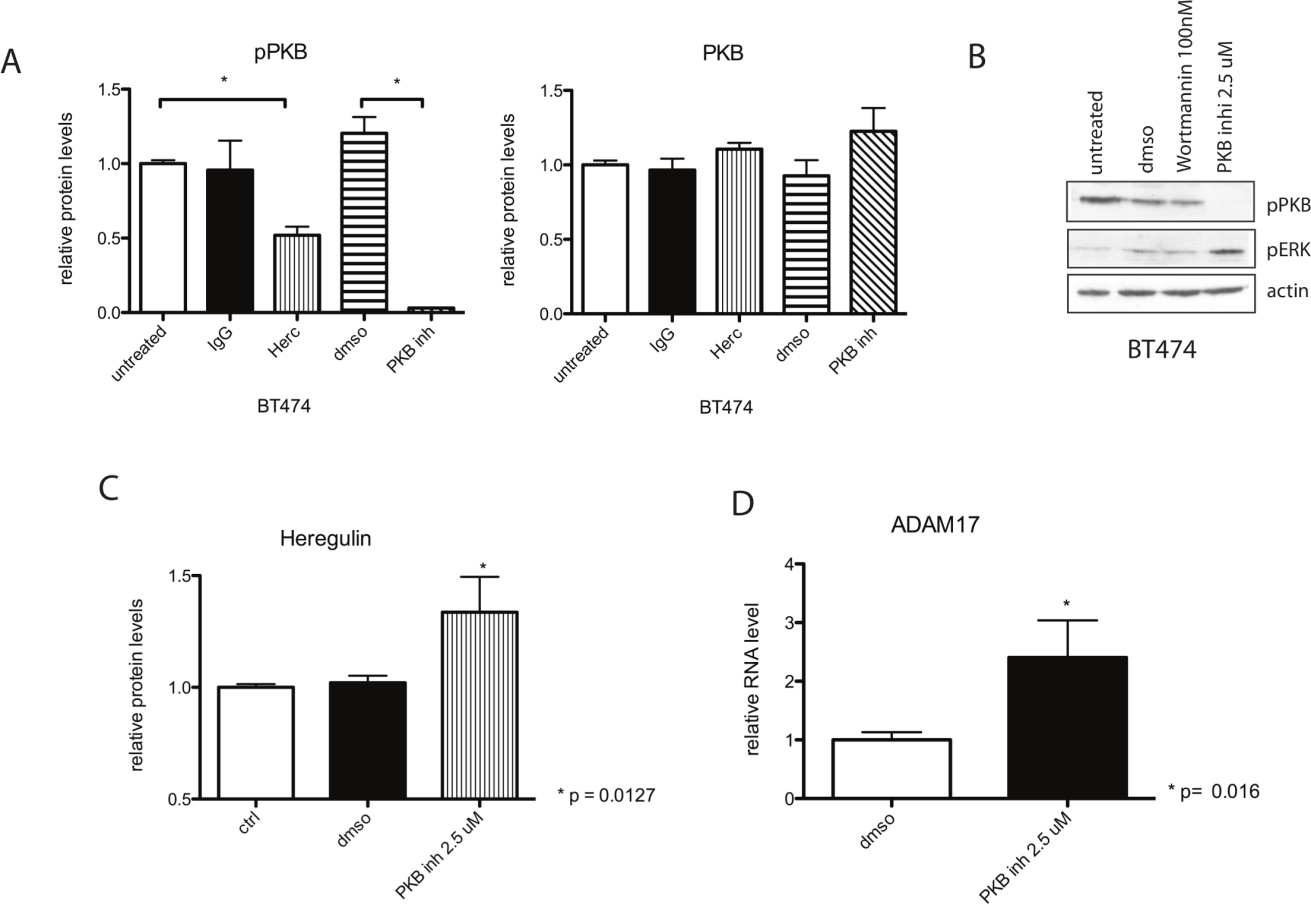
Inhibiting PKB phosphorylation by a PKB inhibitor induces up-regulation of heregulin and ADAM17. (A) BT474 cells were treated for 1 h with 40 μg/ml Herceptin (Herc), 2.5 μM PKB inhibitor (PKB inh), 40 μg/ml IgG control, or DMSO control at 37°C. After 1 h, cells were lysed and analysed for total and phosphorylated PKB levels using MSD multiplex kits. (B) BT474 cells were treated with the indicated treatment conditions for 1 h and the cell lysates were loaded on an SDS gel, and the membranes were probed for the indicated proteins. (C) BT474 cells pre-treated with PKB inhibitor were lysed in Hepes buffer and homogenised before analysing protein levels of heregulin using ELISA. (D) BT474 cells were treated with PKB inhibitor for 1 h, and ADAM17 mRNA levels were studied by QPCR. Statistical significance was determined using the Mann-Whitney test.

**Fig 5:**
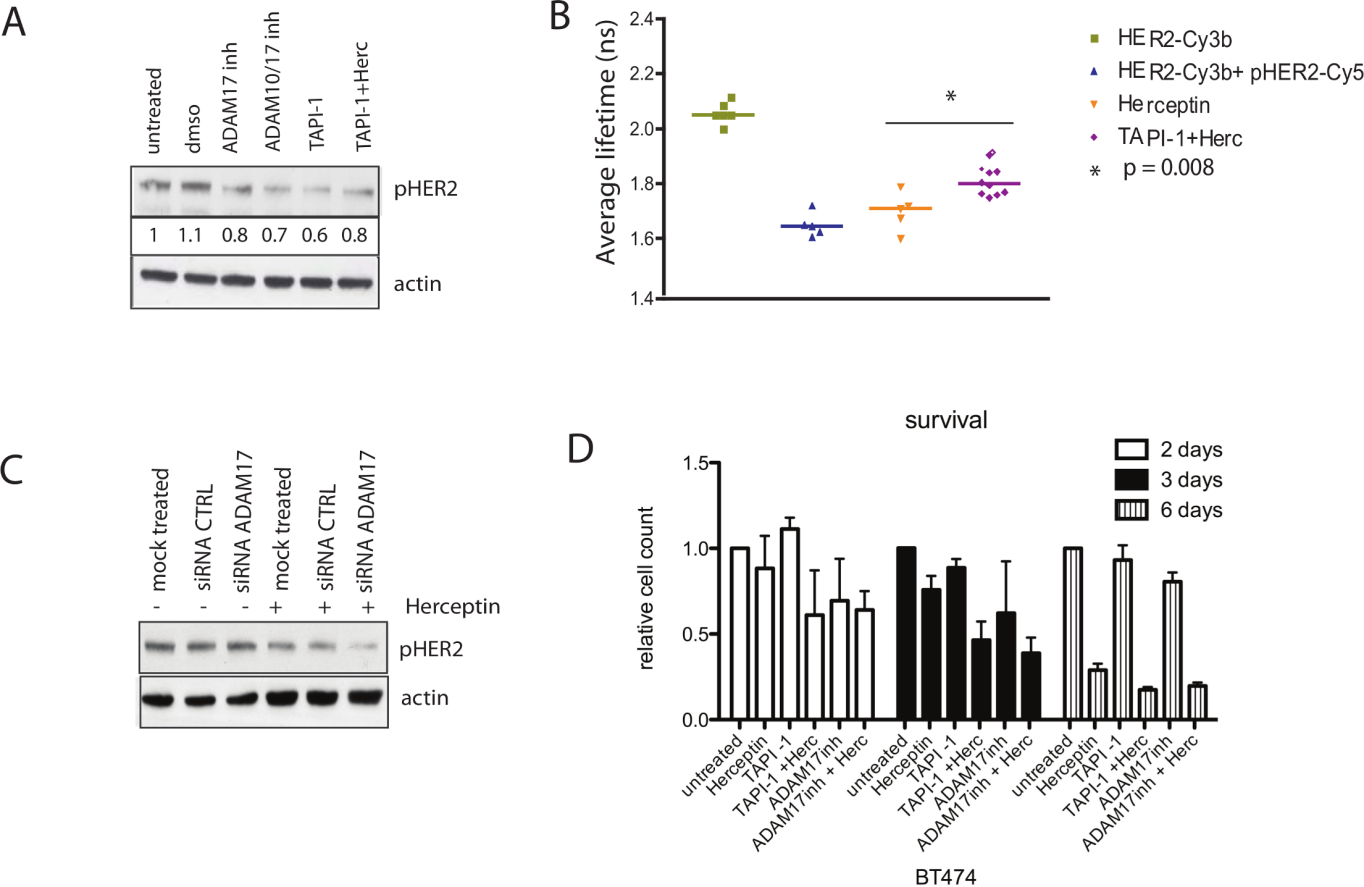
Combination of Herceptin with ADAM inhibitors decreases HER2 phosphorylation and is additive in cell viability inhibition. (A) SKBR3 cells were treated with 10 μM ADAM17 inhibitor (inh), 10 μM ADAM10/17 inhibitor, 10 μM ADAM inhibitor TAPI-1, or a combination of TAPI-1 and 40 μg/ml Herceptin (Herc) for 1 h. Cells were then lysed, and equal amounts of protein were loaded on an SDS gel. Membrane was probed for phosphorylated HER2 and actin. (B) FRET experiments to assess HER2 phosphorylation in SKBR3 cells. The cells were treated with 40 μg/ml Herceptin for 1 h with or without TAPI-1. The medians of the lifetimes were compared with the basal condition using the Mann-Whitney test. (C) Same experiment as Fig 3A; the membranes were probed for the indicated proteins. Only one representative actin control was shown for this experiment. (D) BT474 cells were grown in 24-well plates and left to grow for at least 24 h before being treated for different durations with 40 μg/ml Herceptin, 10 μM TAPI-1, 10 μM ADAM17 inhibitor, or a combination of ADAM inhibitors with Herceptin, as illustrated. The viable cells were counted using a cell counter.
