# Supplementary figures and images for: HER2 Phosphorylation Is Maintained by a PKB Negative Feedback Loop in Response to Anti-HER2 Herceptin in Breast Cancer
Source: PLoS Biol. 2010 Dec 21;8(12):e1000563. doi: 10.1371/journal.pbio.1000563 (PMC3006345; doi:10.1371/journal.pbio.1000563)

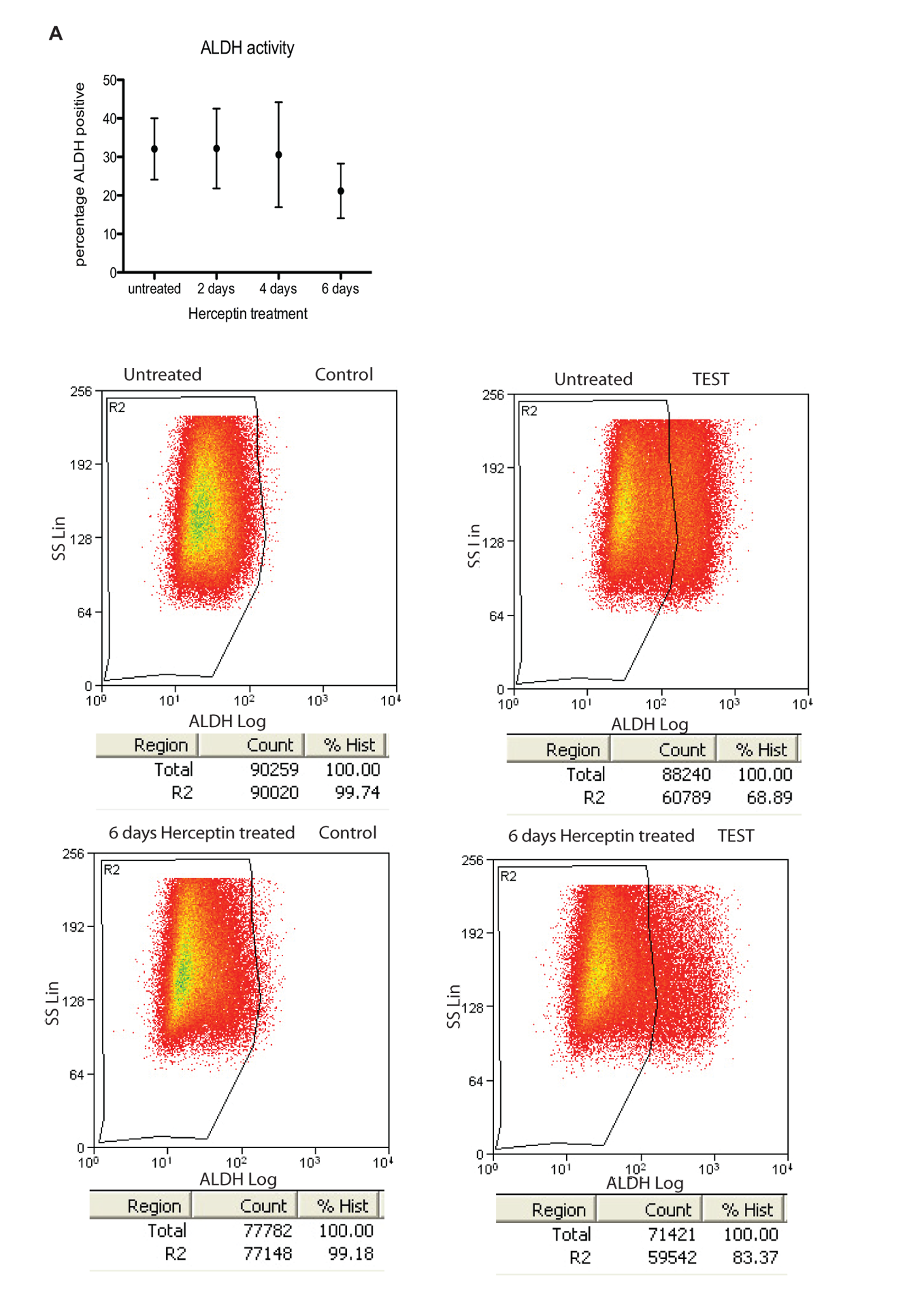

Supplement: Figure S1 — ALDH activity after treatment with Herceptin. SKBR3 cells were treated with 40 µg/ml Herceptin for 2, 4, or 6 d and were then analyzed for percentage of stem cells using the ALDH assay. The experiment was repeated three times, and the average percentage of cells positive for ALDH is depicted in a graph. FACS plots of untreated SKBR3 cells and SKBR3 cells treated with Herceptin for 6 d are depicted. On the left, the control samples are shown, where the ALDH reaction is blocked using DEAB. Negative cells are gated (R2). Using this gate on the right figures, the percentage of ALDH-negative cells can be found. Cells that fall outside of this gate are ALDH positive. (1.31 MB TIF) [file pbio.1000563.s001.tif]

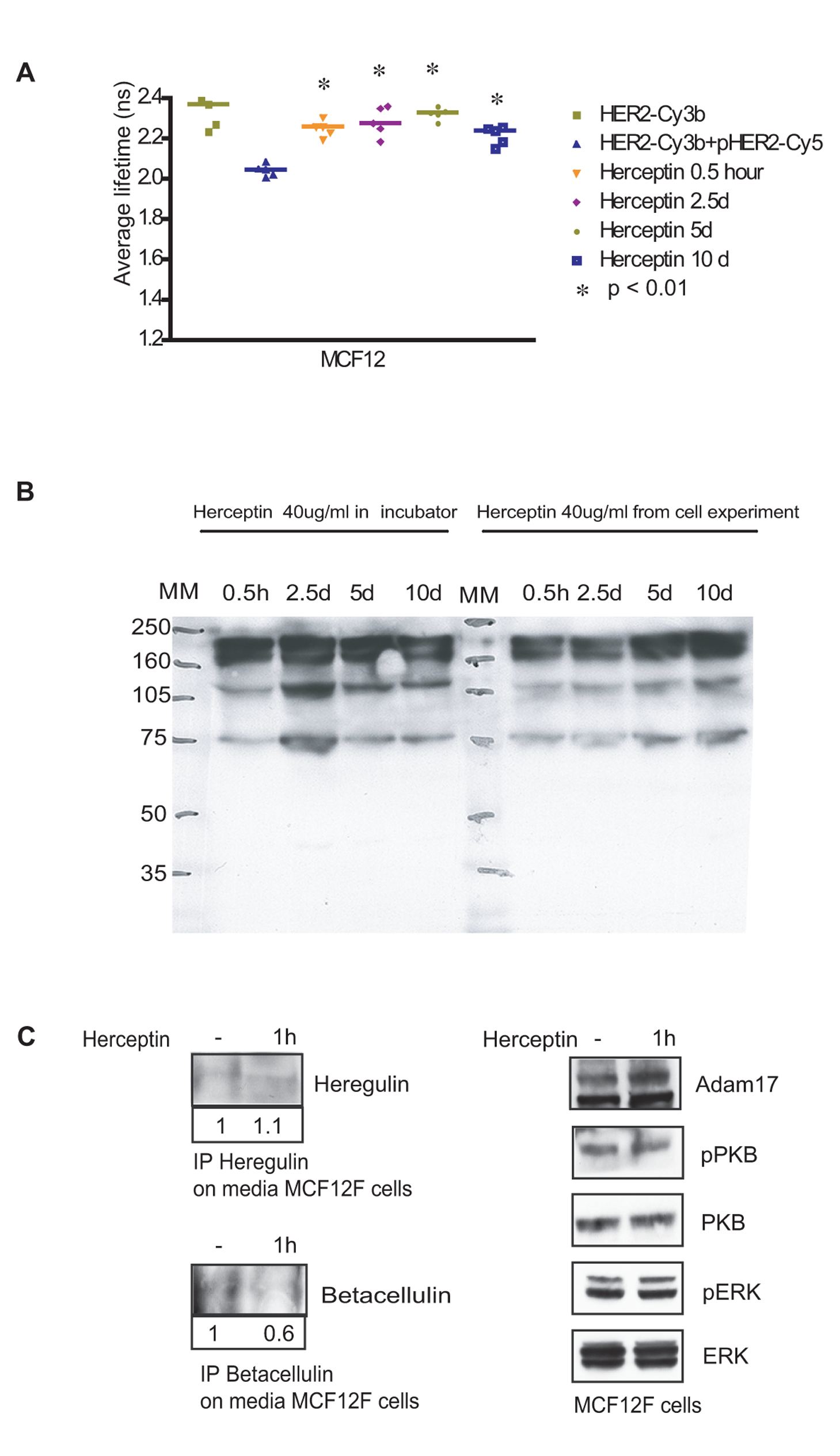

Supplement: Figure S2 — Control experiments. (A) In this experiment, MCF12F cells were incubated with either the donor alone (HER2-Cy3b) or donor and acceptor (HER2-Cy3b+pHER2-Cy5), to assess HER2 phosphorylation by FRET after being pre-treated with different durations of 40 µg/ml Herceptin as illustrated. (B) Western blot experiment using the medium from the SKBR3 cells treated with 40 µg/ml Herceptin as well as medium containing 40 mg/ml Herceptin that was kept in an incubator for up to 10 d. The medium was denatured with SDS-PAGE and boiled for 10 min, and 40 µl of 40 mg/ml Herceptin was loaded in each lane of the SDS-PAGE. The membrane was probed with monoclonal anti-human immunoglobulin antibody that recognises the Fc component of Herceptin. (C) MCF12F cells were treated with 10 µg/ml Herceptin for 1 h in serum-free medium. The medium was analysed for heregulin (left, top panel) and betacellulin (left, bottom panel) using immunoprecipitation. Cells were lysed and analysed by Western blot for ADAM17, pPKB, PKB, pERK, ERK, and actin (right panels). (1.14 MB TIF) [file pbio.1000563.s002.tif]

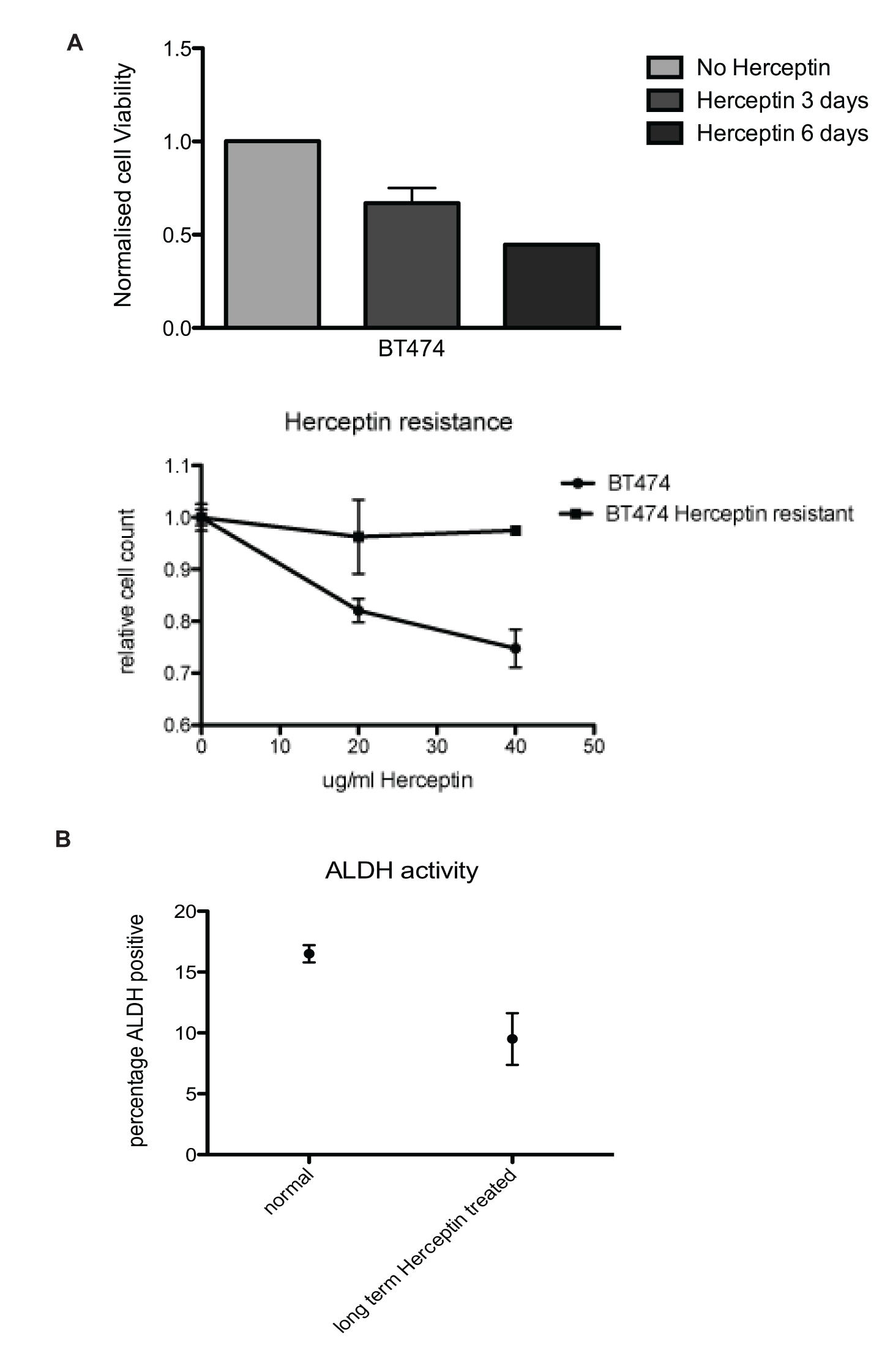

Supplement: Figure S3 — Chronic treatment with Herceptin induces its resistance and decreases ALDH activity in BT474 cells. (A) BT474 cells were treated with 40 µg/ml Herceptin for 3 or 6 d. The cells were trypsinized and counted using a cell counter. In the middle panel, BT474 cells and BT474 cells cultured with 40 µg/ml Herceptin for over 8 mo (Herceptin-resistant BT474 cells) were treated for 6 d with 0, 20, or 40 µg/ml Herceptin. The cells were then trypsinized and counted using a cell counter. (B) Untreated BT474 cells and long-term Herceptin-treated BT474 cells (40 µg/ml Herceptin for over 8 mo) were analyzed using the ALDH assay. The average percentage of cells positive for ALDH is depicted in a graph. (0.26 MB TIF) [file pbio.1000563.s003.tif]

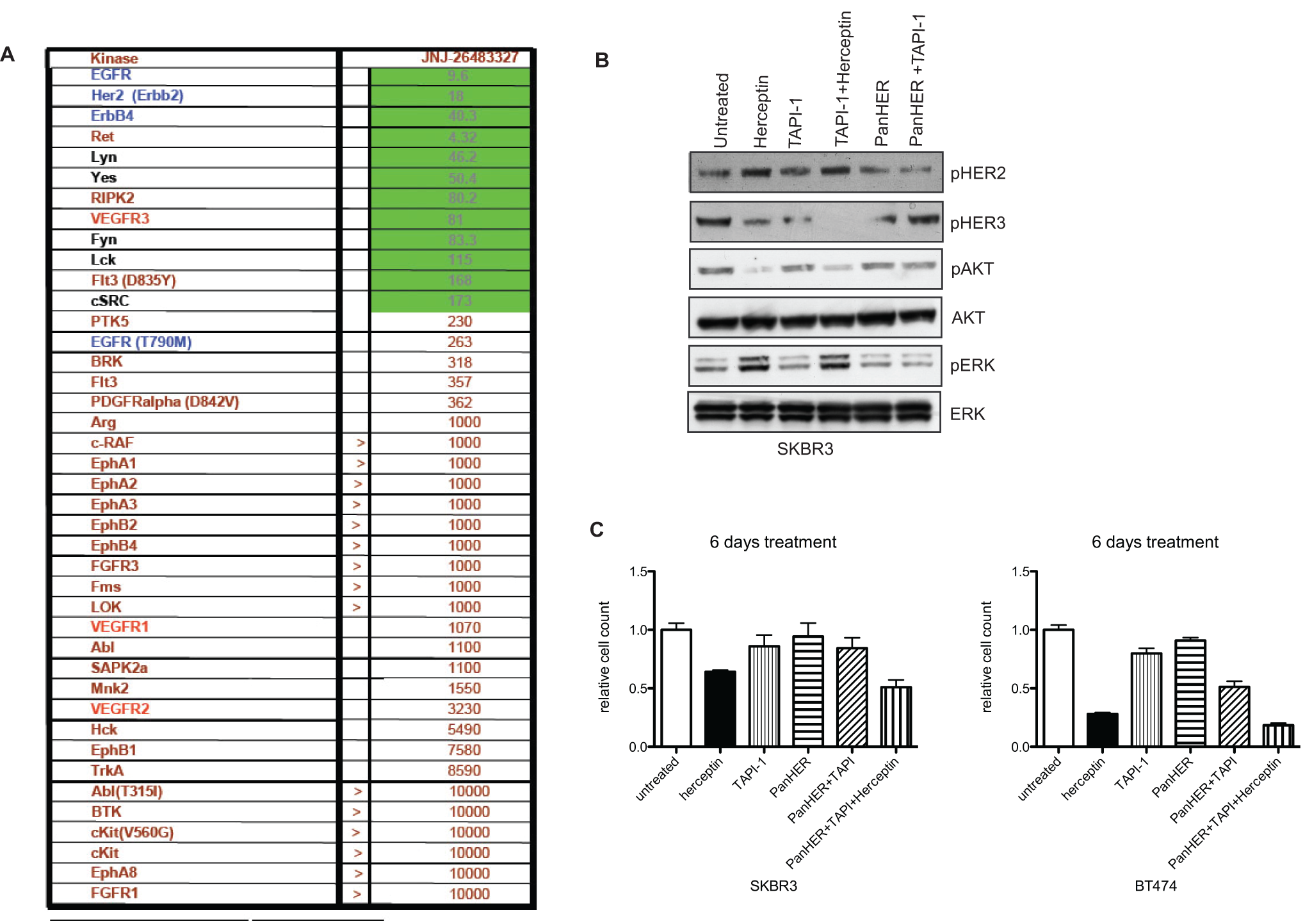

Supplement: Figure S4 — Kinase inhibition assay of JNJ-26483327 and its effect in combination with Herceptin or ADAM17 inhibitor TAPI-1. (A) JNJ-26483327 (a multi-kinase inhibitor and also known as a panHER inhibitor) was tested against a panel of around 230 wild-type and mutant kinases at a concentration of 1 µM and an ATP concentration of 10 µM. Dose response tests were carried out for all those that showed greater than 50% inhibition. (B) SKBR3 cells were treated with 40 µg/ml Herceptin, 10 µM TAPI-1, a combination of TAPI-1 and Herceptin, 5 µM panHER inhibitor (JNJ-26483327), or a combination of the panHER inhibitor with TAPI-1. Western blot analysis was performed for pHER2, pHER3, pPKB, PKB, pERK, and ERK. (C) SKBR3 cells were treated with 40 µg/ml Herceptin, 10 µM TAPI-1, 5 µM JNJ-26483327, a combination of JNJ-26483327 with TAPI-1, or a combination of TAPI-1, JNJ-26483327, and Herceptin for a period of 6 d. The cells were then trypsinized and counted using a cell counter. (1.00 MB TIF) [file pbio.1000563.s004.tif]
